# Ecological costs of climate change on marine predator–prey population distributions by 2050

**DOI:** 10.1002/ece3.5973

**Published:** 2020-01-09

**Authors:** Dinara Sadykova, Beth E. Scott, Michela De Dominicis, Sarah L. Wakelin, Judith Wolf, Alexander Sadykov

**Affiliations:** ^1^ Institute of Biological and Environmental Sciences University of Aberdeen Aberdeen UK; ^2^ School of Biological Sciences Queen's University Belfast Belfast UK; ^3^ National Oceanography Centre Liverpool UK; ^4^ Centre for Ecological and Evolutionary Synthesis University of Oslo Oslo Norway

**Keywords:** Besag, York and Mollie (BYM) models, critical marine habitat, fish, integrated nested Laplace approximation, marine mammals, predator–prey, seabirds, spatial joint modeling

## Abstract

Identifying and quantifying the effects of climate change that alter the habitat overlap of marine predators and their prey population distributions is of great importance for the sustainable management of populations. This study uses Bayesian joint models with integrated nested Laplace approximation (INLA) to predict future spatial density distributions in the form of common spatial trends of predator–prey overlap in 2050 under the “business‐as‐usual, worst‐case” climate change scenario. This was done for combinations of six mobile marine predator species (gray seal, harbor seal, harbor porpoise, common guillemot, black‐legged kittiwake, and northern gannet) and two of their common prey species (herring and sandeels). A range of five explanatory variables that cover both physical and biological aspects of critical marine habitat were used as follows: bottom temperature, stratification, depth‐averaged speed, net primary production, and maximum subsurface chlorophyll. Four different methods were explored to quantify relative ecological cost/benefits of climate change to the common spatial trends of predator–prey density distributions. All but one future joint model showed significant decreases in overall spatial percentage change. The most dramatic loss in predator–prey population overlap was shown by harbor seals with large declines in the common spatial trend for both prey species. On the positive side, both gannets and guillemots are projected to have localized regions with increased overlap with sandeels. Most joint predator–prey models showed large changes in centroid location, however the direction of change in centroids was not simply northwards, but mostly ranged from northwest to northeast. This approach can be very useful in informing the design of spatial management policies under climate change by using the potential differences in ecological costs to weigh up the trade‐offs in decisions involving issues of large‐scale spatial use of our oceans, such as marine protected areas, commercial fishing, and large‐scale marine renewable developments.

## Graphical abstract

This study tests a range of analytical techniques to quantify ecological costs of climate change on habitat availability of predator–prey or competing mobile species. The study demonstrates how to detect environmentally sensitive habitat areas and is highly useful for habitat management policies under climate change.

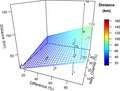

## 1 INTRODUCTION

We need to understand more explicitly the spatial functioning of our marine systems and especially critical marine habitats: those limited areas that are more likely to be the foraging habitats of mobile species such as seabirds and mammals (Cox, Embling, Hosegood, Votier, & Ingram, 2018; Sharples, Scott, & Inall, 2013). This level of understanding is necessary if we are to use marine protected areas (MPAs), marine spatial planning, and changes in spatial management of fisheries effectively (Gaines, White, Carr, & Palumbi, 2010; Gissi, Fraschetti, & Micheli, 2019; Green et al., 2014). Also, to mitigate climate change, we will need to rapidly use more marine space for the large‐scale expansion of marine energy extraction (wind, wave, and tidal), but this will also have ecosystem‐level effects on critical marine habitats (Cazenave, Torres, & Allen, 2016; De Dominicis, O'Hara, & Wolf, 2017; De Dominicis, Wolf, & O'Hara Murray, 2018; Ludewig, 2015; van der Molen, Smith, Lepper, Limpenny, & Rees, 2014). However, the complexity of marine ecosystems is preventing rapid increases in mechanistically detailed knowledge about the entire range of possible changes to the multiplicity of trophic interactions that may occur with both climate change and large‐scale anthropogenic use of our oceans. Therefore, to proceed with spatial planning and management decisions that need rapid answers, we must come up with pragmatic methods that capture the complexity of ecosystems, using what we already know. Also, in order to proceed with some certainty that we are also protecting and maintaining our important top‐predator populations, we need to be able to predict, quantify, and separate the possible “ecological costs” of changes due to climate change from those of large‐scale renewable developments as well as benefits from MPAs. This type of approach will allow the weighing up of trade‐offs in different spatial management decisions (White, Halpern, & Kappel, 2012).

We use the terminology “ecological costs” to cover a range of ecologically important changes to populations, as the purpose of this study was to provide quantitative values of the amount of change to spatial population distributions as well as probabilities of future spatial overlap with prey species. These outcomes can then be used in ecosystem models as predictors of the amount of future population change for a given stressor, that is, climate change or large‐scale renewable developments. In the North Sea and Atlantic region long‐term (up to 40 years) surveys for fish, fish larvae (Edwards et al., 2011; ICES, 2016), seabirds (Kober et al., 2010), and marine mammals (Hammond et al., 2013), as well as more recent use of tagged mammals and seabirds (Jones et al., 2013; Wakefield et al., 2017), have allowed the creation of seasonal and annual spatial distributions of either density or abundance depending on the data for the mobile species (see section on “Study Area and Species”). Therefore, we know a lot about “where” seabirds and mammals and some of their main prey species may be located but we know far less about exactly “why” they are there, for example, what it is about these habitats that make them the (potentially limited) areas where mobile animals forage (Cox et al., 2018). This study sets out to investigate the use of a range of analytical techniques to produce outputs that can be used to assess the spatial changes to populations that we are calling “ecological costs” of climate change, on the locations where highly mobile marine predators capture prey and where mobile competing species may overlap. This study uses spatial analytical tools (Sadykova et al., 2017) that can estimate the degree and strength of overlap (referred to as “common spatial trends”) of spatial locations driven by important biological and physical variables by using a Bayesian hierarchical joint modeling approach with integrated nested Laplace approximation, INLA (Rue, Martino, & Chopin, 2009; Rue et al., 2017). The “common spatial trend” quantifies the degree of spatial overlap and specifically is the posterior residual spatial autocorrelation unexplained by covariates.

We explore spatial joint model outputs to compare the efficiency of methods that estimate the ecological costs of trophic interactions between predator and prey and competing species for similar prey, in present versus future “business‐as‐usual” (worst‐case) greenhouse‐gas emissions scenario (De Dominicis et al., 2018; Stocker et al., 2013), projected to mean climate change conditions centered on 2050. We used the “worst‐case” scenario in order to calculate the maximum ecological cost. We ran the models up to 2050 as it is only after this time period that the effects of different scenarios start to become more important (IPCC, 2018). Therefore, in this study we are comparing the current situation to what the marine environment will most likely look like in 2050.

We use a selected group of important biological and physical variables (see section “Physical Environmental Variables” for more details) that will change with climate change and have been shown to be important to marine mammals and seabirds and their prey (Carroll et al. 2015, Chavez‐Rosales, Palka, Garrison, & Josephson, 2019, Sadykova et al., 2017, Wakefield et al. 2017). The three aims of this study are first to compare joint model distributions of a range of contrasting seabird and mammal species from the present to future (2050) climate change projections. The second aim is to quantify estimates of the changes in spatial population density or abundance distributions, which we are defining as ecological costs/benefits, by four different methods. The third aim is to test whether some or all of the methods show the same degree and direction of change, to determine which method or combination of methods is the most effective predictor of both quantitative and spatial changes. The four methods include both single‐species and joint model outcomes. Method 1 is the prediction of the amount of relative change in the populations for single species for each of the joint modeling future scenarios. Method 2 is assessing the overall spatial change in either density or abundance for single‐species models and common spatial trends of the joint models by quantifying the number of grid cells within the study area that shows >33% change in value. This method is called overall spatial percentage change. Method 3 uses outcomes of the percentage of grid cells that show either a loss or gain of habitat (for single‐species models) and a change from either positive to negative or vice versa in the common habitat trends (for joint models). Method 4 quantifies the distance between weighted centroids of each joint model and the direction of travel between the present and future common habitat trends.

## 2 DATA DESCRIPTION

### 2.1 Study area and species

The study area was defined as covering the North Sea and the UK continental shelf in the area between 48° and 62° north and 10° west and 10° east. We used spatial distributional data on eight mobile marine top‐predator species: gray seal (*Halichoerus grypus*), harbor seal (*Phoca vitulina*), harbor porpoise (*Phocoena phocoena*), common guillemot (*Uria aalge*), black‐legged kittiwake (*Rissa tridactyla*), northern gannet (*Morus bassanus*), herring (*Clupea harengus*), and sandeels (*Ammodytidae*). The seabird and mammal species were chosen to provide contrasts in their foraging and breeding behaviors and for the high level of spatial and temporal data availability. Within the seabirds, kittiwakes represent surface feeders, guillemots—deep divers (>150 m), and gannets—plunge divers of intermediate depths (~30 m). Gray and harbor seals were selected due to their similarities in diet and foraging behaviors and yet contrasting population dynamics: Gray seal populations are currently rapidly increasing and harbor seals declining (Wilson & Hammond, 2019). Harbor porpoise were selected as the most common cetacean species (Hammond et al., 2013). For fish prey species, we choose two species that are very common in the diet of all the above top predators (Booth, 2019; Wanless, Harris, Newell, Speakman, & Daunt, 2018; Wilson & Hammond, 2019) but have very contrasting behaviors and habitat use. Sandeels have very specific benthic habitat needs (Wright, Jensen, & Tuck, 2000) with localized populations that have a tendency to remain in the same areas throughout their life histories (van der Kooij, Scott, & Mackinson, 2008; Wright, Régnier, Gibb, Augley, & Devalla, 2018). Herring, in particular juvenile herring, have large larval movements (age 1) and adult annual migrations (age 2,3 and older), such that they are found in quite different habitats at different ages and seasons (Corten, 2002). For all present climate distributions of single species, see Figures S1.1a–S1.9a, the left panel of each figure.

The gray and harbor seal usage maps represent estimated annual at‐sea density distributions of seals over 20 years from surveys and tagging programs (Jones, McConnell, Sparling, & Matthiopoulos, 2013). The harbor porpoise density maps represent porpoise density from whole UK surveys in 1994 and 2005 (Hammond et al., 2013), and unique from all the other data used in this study, these outputs are model estimates of density distributions based on environmental covariates. They were created by combining the outputs of a smooth surfaces generated from species distribution models using latitude, longitude, and depth in 1994, and using latitude, longitude, depth, and distance to coast in 2005 (Hammond et al., 2013). The potential issues and care that must be given with the interpretation of these results will be examined in the results and discussion. Maps of the herring abundance represent the herring mean abundance from summer acoustic surveys for juveniles (age 1) and all older age classes (2 and 3+) for the combination of the years: 2003–2009 and 2013–2014 provided by the Herring Assessment Working Group (HAWG, 2019), (ICES, 2015, 2016). The common guillemot, black‐legged kittiwake, and northern gannet density maps show seabird density across 25 years (1989–2014) of data from at‐sea surveys with data from all seasons (Kober et al., 2010). The density maps for sandeels represent larval density across 25 years (1989–2014) from continuous plankton recorder (CPR) data (Edwards et al., 2011). All the species data were given on a regular 7 × 7 km square grid. All the data points with depth >500 m were removed from consideration as the prey species of focus in this study all live in habitats that do not go beyond those depths. More details about the methods of modeling the different species can be found in Sadykova et al. (2017).

### 2.2 Physical environmental variables

Data on five biophysical environmental variables have been provided from runs of the Atlantic Margin Model 7 × 7 km (AMM7‐NEMO) 3D baroclinic, hydrodynamic model, coupled with an ecosystem model ERSEM (Wakelin, Artioli, Butenschön, Allen, & Holt, 2015). The variables used are as follows: bottom temperature (BT) (°C), maximum chlorophyll‐a (CHL) (mgC/m^3^), net primary production (NPP) (gC m^2^ year^−1^), potential energy anomaly (PEA, which is the energy required to mix the water column completely) (J/m^3^), and depth‐averaged current speed (SP) (m/s). The variables were used in two seasons: “spring” and “summer,” and this is described in the following section. These variables were chosen as they cover the main physical and biological parameters that can affect pelagic habitats and primary production (Holt, Butenschon, Wakelin, Artioli, & Allen, 2012; Holt, Hughes, et al., 2012; Holt & Proctor, 2008; Holt et al., 2016) under both climate change and, the next biggest change to our shallow seas, very large extraction of energy from offshore renewable developments (Boon et al., 2018; De Dominics et al., 2018; van der Molen et al., 2014). These variables are important habitat variables as they capture the range of features: fronts, other areas of high production, and mixing characteristics of shallow seas including density differences due to regions of freshwater influence (Cox et al., 2018). PEA is a continuous variable that captures the main vertical mixing characteristics (level of stratification) of the water column, such as differences in pycnocline gradient and depth and positions of fronts, and will generally increase with distance to shore, as the value of depth increases (Holt & Proctor, 2008). BT is similar to PEA, in fact the two variables are highly correlated (*r* > .6) such that both will never be run in the same model. However, PEA will change more with climate change via changes to stratification by surface warming, whereas BT will not increase as much (De Dominicis et al., 2018). Therefore, BT was also used to cover any physiological responses, especially from prey and mammals where temperature may play a more important role than stratification. SP was used to pick up areas where predators use different horizontal current speeds (Benjamins et al., 2015). The two biological variables NPP and CHL are not correlated in the summer  season (*r* < .2). High levels of NPP represent different areas in the spring versus summer seasons. In spring, they can represent anywhere there is a spring bloom occurring, whereas in the summer they will mostly represent areas where the whole water column is mixed, normally near shore, shallower regions (Holt et al., 2016). CHL represents much more fine‐scale areas of higher localized production due to internal waves at shelf edges, banks, and troughs (Cox et al., 2018). A range of studies have shown their importance for specific seabird and marine mammal species (Van Beest et al., 2019; Bost et al., 2009; Carroll et al., 2015; Chavez‐Rosales et al., 2019; Scott et al., 2010; Waggitt et al., 2018; Wakefield et al., 2017).

All the variables were on a regular 7 × 7 km square grid for 2 seasons: The first season (“spring season”) represents spring and early summer and includes March, April, May, and June and the second season (“summer season”) includes July, August, September, and October. Having the data in two seasons allows capture of the major differences in availability of prey (sandeels, mostly available only in the spring season, and herring, available during both seasons but with different distributions) and the spatial foraging changes due to differences in breeding (central‐placed) and postbreeding behaviors of at least the seabird species. As the distribution for seals was from annual usage maps (Jones et al., 2013), both seasons are represented, and with porpoise data derived during July, their distribution represents only the summer season (Hammond et al., 2013). All the data were given as climatological means across 25 years for present (1989–2014) and future (projected) (2037–2062) years, using a single climate scenario termed the “business‐as‐usual” or “worst‐case” climate scenario (Stocker et al., 2013). AMM7‐NEMO was used to downscale the climate signal from the Hadgem2‐ES climate model (The HadGEM2 Development Team, 2011). Present and future outputs for each variable are presented in Figures S2.1–S2.5.

## 3 METHODS

### 3.1 Single‐species and joint models

We used Besag, York, and Mollie (BYM) (Besag, York, & Mollie, 1991) spatial hurdle and nonhurdle single and joint models following Sadykova et al. (2017). BYM is an intrinsic autoregressive model, where the spatial effect of a particular area depends on the effects of the neighboring areas. In addition, the BYM specification allows inclusion of a heterogeneous effect, which assumes that the obtained estimates between areas are independent of each other (Besag et al., 1991). Both spatial effect and heterogeneous effect are referred to as “random effects” in this paper. The hurdle models, which have been developed to manage the high occurrence of zeros in the observed data, were considered for data with an excess of zeros (harbor seal, sandeels, and common guillemot), while nonhurdle models were considered for the data without excess of zeros. A hurdle model is a two‐component model, where the first part presents a binary component that generates zeros and ones (0—zero values, 1—positive values). The second part of the hurdle model presents an amount component that generates nonzero values (for positive density or abundance). The “hurdle” value (which may present any value) was set at zero in this study. Gamma likelihood was assumed for the positive density or abundance data, and logistic regression was used for the binary process in hurdle models. For the data without excess zeros, we assumed a model with gamma likelihood (Sadykova et al., 2017).

The parameters were modeled as sums over different combinations of the biophysical variables' effects (we considered all possible combinations of the covariates excluding highly correlated (*r* > .5) variables) and the random effects, due to unstructured and spatially structured heterogeneity. The covariates' effects were modeled as smooth functions of either first‐order or second‐order random walk processes to pick up smooth fluctuations (Rue & Held, 2005). For a detailed description of the models, readers are referred to Sadykova et al. (2017). The Bayesian hierarchical modeling approach with integrated nested Laplace approximation (INLA) was applied to reduce the computational cost of fitting these spatial models (Rue et al., 2009).

### 3.2 Joint model selection

Joint‐species models were considered, in order to select the best habitat models for coupled species (e.g., predator–prey or competing species). The coupled species were assumed to share the same biophysical habitat variables (find more about single‐species habitat selection in Sadykova et al., 2017). We considered all possible combinations of covariates (biophysical variables), excluding the combinations with highly (*r* > 0.5) correlated variables (BT was strongly correlated with NPP and PEA, NPP was highly correlated with CHL). The goodness of fit for all the joint‐species models with all the considered combinations of covariates was assessed using the deviance information criterion (DIC; Spiegelhalter, Best, Carlin, & Linde, 2014). The models with the lowest DIC values were considered as the best models. All computations were performed using the R‐INLA package (Lindgren & Rue, 2015; Rue et al., 2009, 2013).

### 3.3 Single‐species and joint model predictions

Integrated nested Laplace approximation provides predictions of missing values in the response. We used this feature to provide predictions of the density values using future climate scenario biophysical data (2037–2062). Specifically, making a prediction for a response variable in INLA is the same as fitting a model with some missing data. The response values (abundances or densities) that we want to predict are set to be “NA” (not available) for all the locations. Then, the models were fitted using both the present biophysical variables (for the present abundances or densities values) and the projected future climate scenario biophysical variables (2037–2062) (for the missing values). The BYM predictive models also included the random effects, due to unstructured and spatially structured heterogeneity (for more details, see “Single‐species and joint models” subsection). The biophysical covariates for each model were selected based on the model selection results.

### 3.4 Methods for analyzing spatial differences: estimates of ecological costs

Four methods were used to assess differences between present and future populations and spatial distributions of single‐species and joint‐species model outcomes. These differences are used to compare the estimates of potential ecological costs of climate change to gauge whether each of the four methods for ecological costs indicates the same magnitude and/or direction of change. Note for Method 3, the local and common spatial percentage loss/gain calculations have separate methods for single‐species versus joint models (see subsection “Local and Common Space Percentage Loss or Gain” below).

#### 3.4.1 Relative population change: Method 1

The first method assessed predicted relative changes in populations for each single‐species and joint model. This was accomplished by comparing present (1989–2014) and future (projected) (2037–2062) densities for each predictive model. The outputs of this analysis are used for each of the following three methods for quantifying ecological costs.

#### 3.4.2 Overall spatial percentage difference: Method 2

The comparisons of overall spatial percentage difference in density or abundances, between future and present single‐species and joint model common spatial trends, were made by evaluating the percentage change for each grid cell and then also estimating the percentage of the entire area (over all grid cells) that exceeds the one‐third difference (i.e., >33% of all grids are different). The “thirds rule of thumb” (Berry, 2007) says that if two‐thirds of the total area (67%) has grid cells with >33% differences, the surfaces are “fairly different,” otherwise the future distributions are “fairly similar” (Berry, 2007). In this study, we have introduced three categories. The future distributions are (1) “fairly similar,” when <33% of the total area is different (has grid cells with >33% differences) from the present common spatial trend; (2) “fairly different,” when the difference is in the range 34%–66%; and (3) “very different” when >67% of the total area is different. Analysis considered in this paper included all grid points (following Berry, 2007), to take all spatial information into account. However, we also calculated spatial percentage difference with three different thresholds to find out whether small values are influencing the percentage difference outcomes and estimating percentage differences in the highly populated habitat areas.

#### 3.4.3 Local and common space percentage loss or gain: Method 3

##### Single‐species models: Percentage of local area loss or gain

To explore local patterns in the decrease or increase in densities of single‐species future (projected) distributions, we defined “hot spots” and “cold spots” using G* statistics by Getis and Ord (1992). Percentage of the population change (decrease/increase) was estimated as the percentage of the grid cells where “hot spots” moved to “cold spots” (decrease, lost area) or moved from “cold spots” to “hot spots” (increase, gained area).

##### Joint models: Percentage of local area loss/gain in common spatial trends

Percentage of common spatial trend (joint habitat) loss was calculated as the percentage of the grid points that are not going to be suitable for both predator and prey or competing species in the future; in other words, the grid points where the common spatial trend changed sign from positive to negative. Conversely, the percentage of new suitable common habitat was estimated as the percentage of the grid points where the spatial trend changed sign from negative to positive. This analysis considered only those grid‐point values in which 95% credible intervals included either only negative or only positive values (i.e., significant). The analysis sums up the number of grid cells where the common spatial trend will have changed sign from negative to positive (gain overlap) or positive to negative (lost overlap) and calculates the percentage of the present common trend area that will change in the future 2050 scenario.

#### 3.4.4 Weighted centroids and direction of change: Method 4

To estimate the effect of climate change on the species distributions, we calculated differences between weighted centroids of the present and future (projected) populations of both single‐species and joint model outputs. Each weighted centroid represents how the population is spatially distributed, summarized as a single reference point on the map, and was calculated using a mean centroid algorithm (first moment of area; Kumler & Goodchild, 1992; Tukey, 1977). Differences between weighted centroids of the present and future population densities and present and future common habitat trends (based on the positive values) were used to estimate the effect of climate change on both densities and the common species habitat and were calculated using the haversine formula (Inman, 1835). The direction of travel was obtained as the orientation between two centroids (reference points). As population densities were predicted from different models, with different biophysical covariates, a range of differences between weighted centroids was obtained.

### 3.5 Comparisons with other common methods: RMSE and Bhattacharyya distance

We compared the results from our research with the results that would be obtained using two other common statistical methods. The first method is root mean square error (RMSE), also known as root mean square deviation, which is commonly used to compare two data sets (Hyndman & Koehler, 2006). Here, we used it to compare future (projected) and present common spatial trends to estimate relative ecological costs of climate change (note that we did not use the RMSE method to evaluate our models, but to compare two different map surfaces). The RMSE formula that we used was RMSE = $\sqrt{\frac{\sum_{i=1}^{N}{\left({\text{Future}}_{\text{CST}}-{\text{Present}}_{\text{CST}}\right)}^{2}}{N}}$, where *N* is a sample size and Future_CST_, Present_CST_ are future (projected) and present common spatial trends. The second method that we used is estimating Bhattacharyya distances (Bhattacharyya, 1943), which reflect the degree of dissimilarity (also called “measure of divergence”) between the future (projected) and present common spatial trends.

### 3.6 Percentage of deviance explained

To estimate the percentage of the null deviance explained by each model predictor (each biophysical variable), the following formula has been used:

$$\frac{\left(\text{Null deviance}\;-\text{Reduced model deviance}\right)}{\text{Null deviance}}\times 100.$$

where the null deviance is a deviance of a model that includes only the intercept (“worst model”) and reduced model deviance is a deviance of the reduced model with only one biophysical variable. This diagnostic measure is used to indicate the explanatory power brought individually by each biophysical variable. This measure can also indicate the potential level of bias in the outputs including the porpoise density data, which had estimated density surfaces based on correlations with depth and distance to coast (see “Data description” section).

## 4 RESULTS

### 4.1 Single‐species model selection

Overall, outcomes of both single and joint models showed that PEA and NPP are the most important habitat‐proxy variables, with CHL being the 3rd most important.

The direction/shape of the relationship for single‐species model selection results is shown in Table 1. As there were some models with 2 or less DIC points between them, the 2nd best models were also presented. Across species, PEA was the most important variable in 9 out of 14 models (7 out of 8 species) with NPP the next with 8 appearances (7/8 species) and CHL with 6 (5/8 species). The PEA relationships were almost always negative across species; however, for herring age 2+3, the relationship contains an optimal value for PEA. The CHL relationships are mostly positive and have an optimum with porpoise, but again, herring aged 2+3 is the exception with a negative relationship with CHL. NPP, SP, and BT all had very variable relationships with different relationships between the different species. The percentage of deviance explained by each biophysical variable can also be seen in Table S3, which confirms that PEA is the variable that explains the highest deviance in 5 of the species and the combination of PEA, NPP, and CHL are consistently the top 3 variables for 7 out of 8 species. The Table S3 also shows that the biophysical variables for the porpoise models do not have the higher explained deviance than those of the other species, which provides support in using the biophysical variables for the porpoise density surfaces.

**Table 1:** Deviance information criterion (DIC)‐based single‐species model selection results

Model	BT	CHL	NPP	PEA	SP
(1) Gray seals			asm	neg	
(1) Harbor seals		pos		neg	
(2) Harbor seals			pos	neg	
(1) Porpoises	opt	opt			neg
(2) Porpoises			opt	neg	
(1) Northern gannet			opt		opt
(1) Common guillemot		pos		neg	
(1) Black‐legged kittiwake		pos		neg	
(2) Black‐legged kittiwake			pos	neg	
(1) Herring age 1			opt		pos
(2) Herring age 1		pos			pos
(1) Herring age 2+3	neg	neg			
(2) Herring age 2+3			opt	opt	
(1) Sandeels			neg	neg	

Note

The best‐supported models are shown for each species (number (1)), and variables included in the best models are shaded in blue. When the difference in DIC between the best model and the next best model is less than 2, the second best models are reported (number (2)) and variables included in the 2nd best models are shaded in gray. Selected models for herring are given for different age‐groups (age 1 and ages 2 and 3). The biological and physical variables are as follows: bottom temperature (BT), maximum chlorophyll‐a (CHL), net primary production (NPP), potential energy anomaly (PEA), and depth‐averaged current speed (SP). “Pos” indicates a positive relationship, “neg”—a negative relationship, “opt”—that the relationship contains an optimal value, and “asm”—that the relationship is asymptotic.

Note, due to a range of improvements, including updated input data from the NEMO‐ERSEM data sets, limiting the analysis to depths of <500 m (as prey species are not found beyond those depths) and the use of predictive modeling in this study, the single‐species model results are different from those of Sadykova et al. (2017).

### 4.2 Joint model selection and common spatial trends

**Figure 1:**
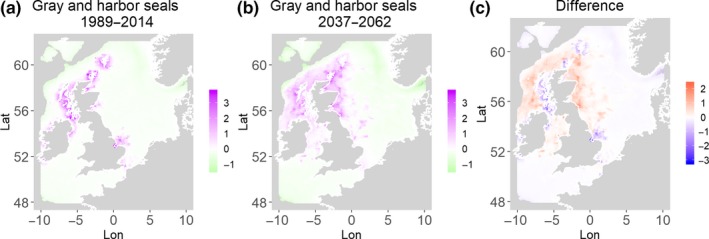
Gray seals and harbor seals. Estimated present common spatial trend (1989–2014) (a) and future (projected) common spatial trend using future climate scenario data (2037–2062) (b). The right picture (c) shows difference between the future and the past common spatial trends

**Figure 2:**
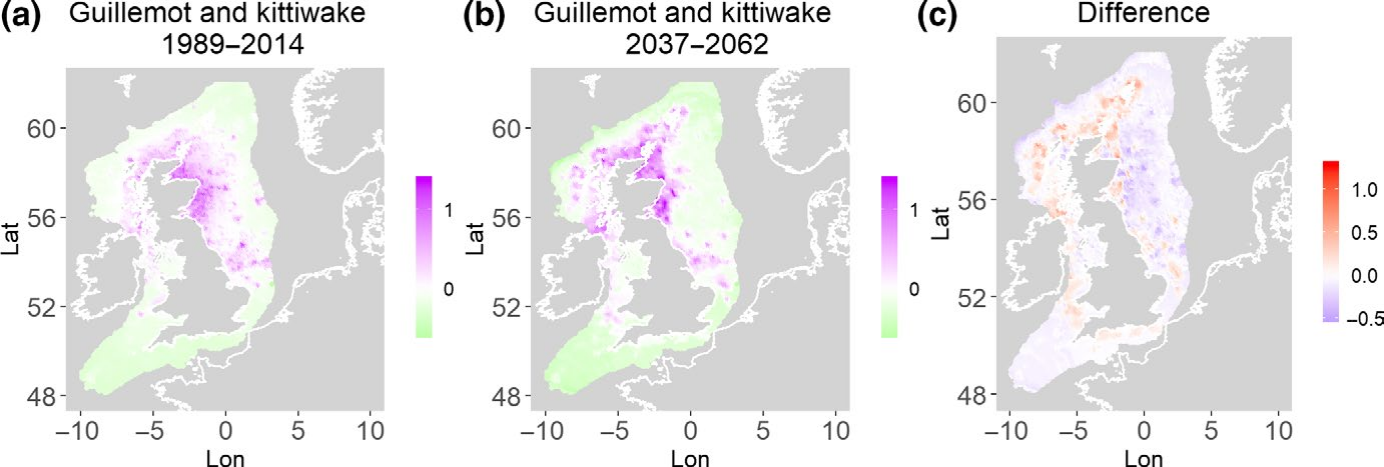
Common guillemot and black‐legged kittiwake. Estimated present common spatial trend (1989–2014) (a) and future (projected) common spatial trend using future climate scenario data (2037–2062) (b). The right picture (c) shows difference between the future and the past common spatial trends

**Figure 3:**
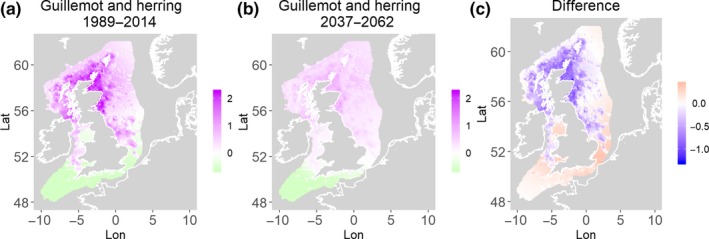
Common guillemot and herring. Estimated present common spatial trend (1989–2014) (a) and future (projected) common spatial trend using future climate scenario data (2037–2062) (b). The right picture (c) shows difference between the future and the past common spatial trends

**Figure 4:**
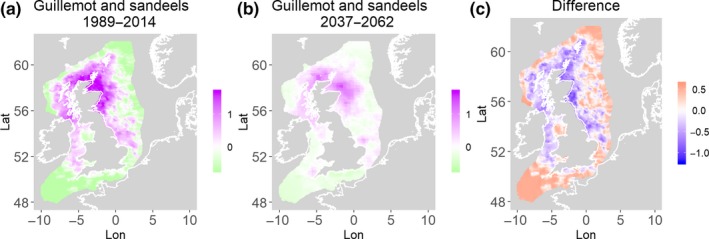
Common guillemot and sandeels. Estimated present common spatial trend (1989–2014) (a) and future (projected) common spatial trend using future climate scenario data (2037–2062) (b). The right picture (c) shows difference between the future and the past common spatial trends

**Figure 5:**
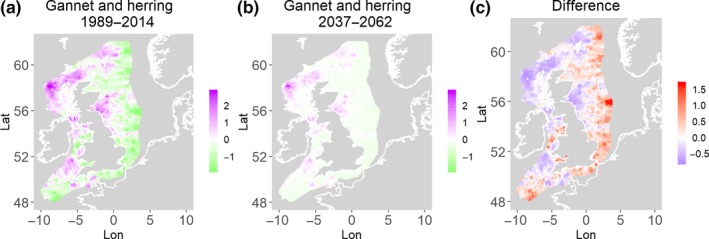
Northern gannet and herring. Estimated present common spatial trend (1989–2014) (a) and future (projected) common spatial trend using future climate scenario data (2037–2062) (b). The right picture (c) shows difference between the future and the past common spatial trends

**Figure 6:**
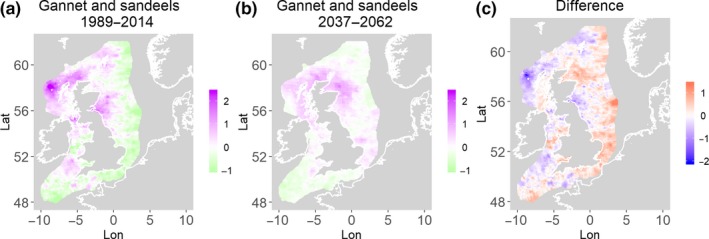
Northern gannet and sandeels. Estimated present common spatial trend (1989–2014) (a) and future (projected) common spatial trend using future climate scenario data (2037–2062) (b). The right picture (c) shows difference between the future and the past common spatial trends

**Figure 7:**
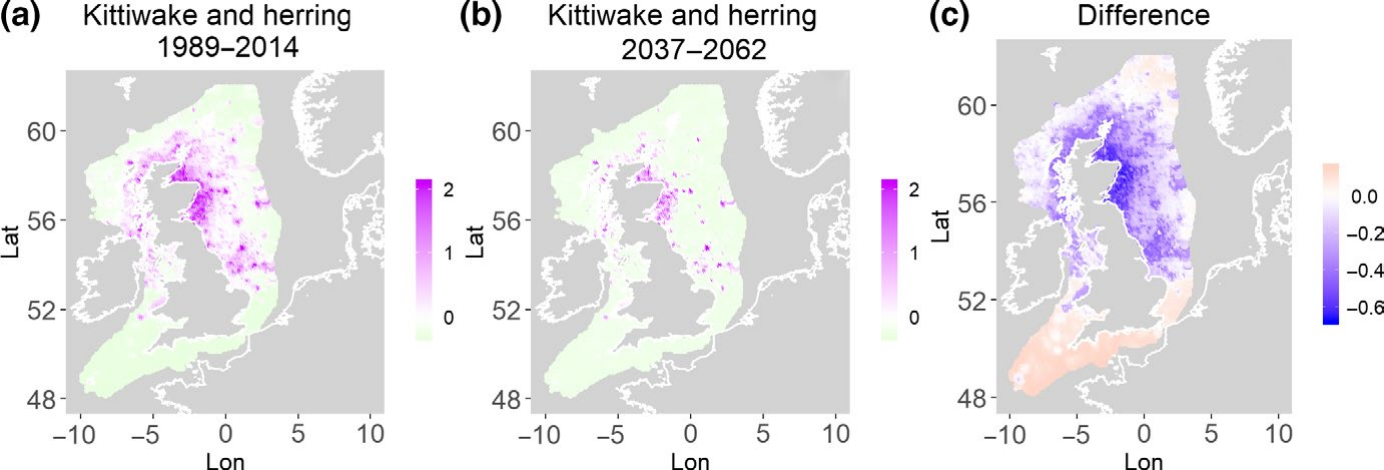
Black‐legged kittiwake and herring. Estimated present common spatial trend (1989–2014) (a) and future (projected) common spatial trend using future climate scenario data (2037–2062) (b). The right picture (c) shows difference between the future and the past common spatial trends

**Figure 8:**
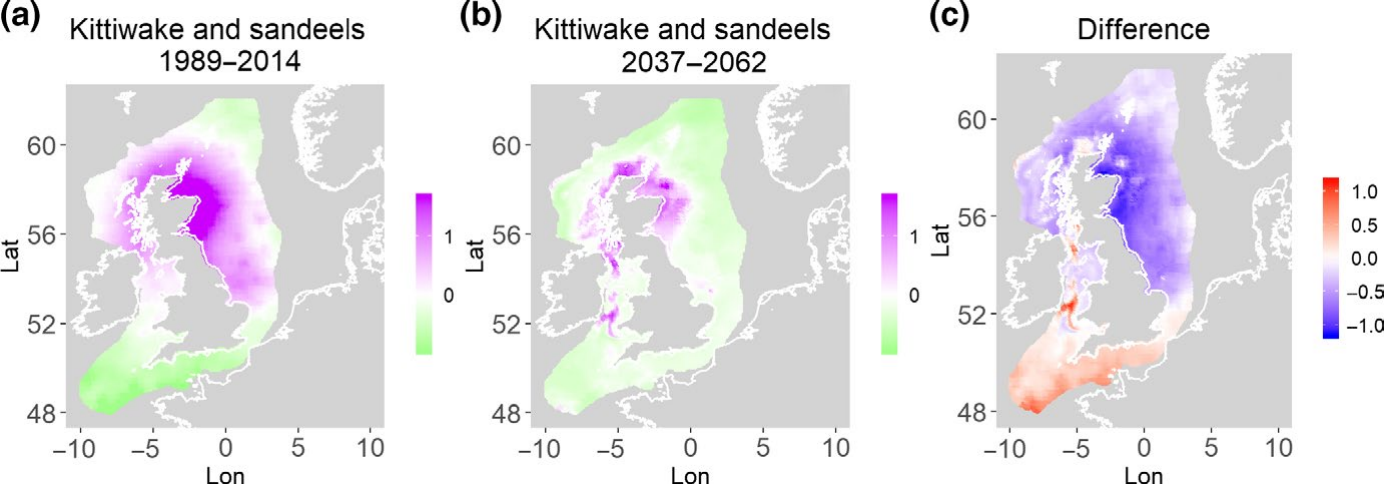
Black‐legged kittiwake and sandeels. Estimated present common spatial trend (1989–2014) (a) and future (projected) common spatial trend using future climate scenario data (2037–2062) (b). The right picture (c) shows difference between the future and the past common spatial trends

**Figure 9:**
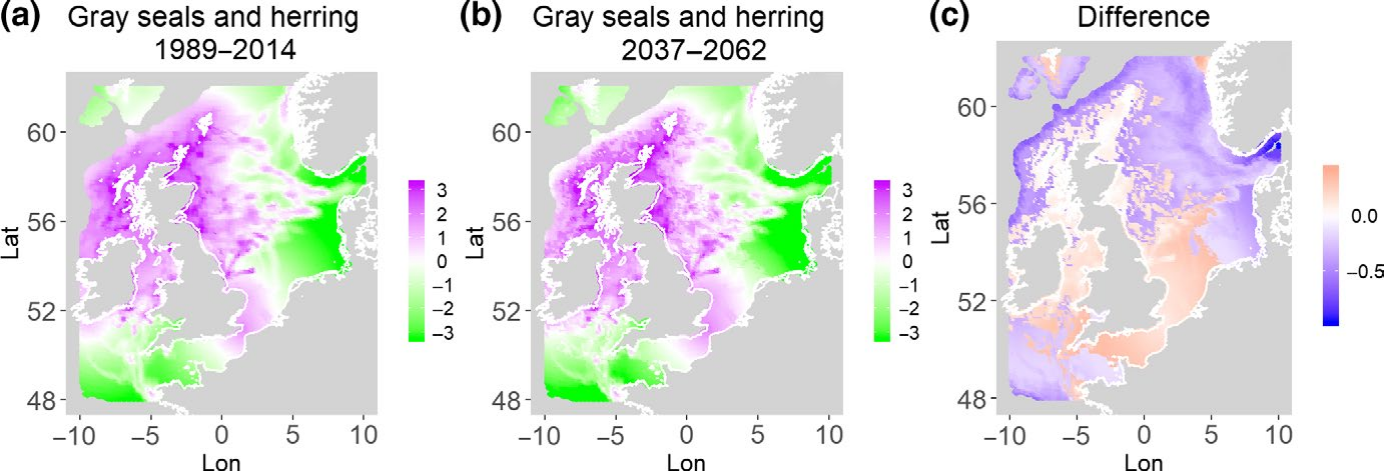
Gray seals and herring. Estimated present common spatial trend (1989–2014) (a) and future (projected) common spatial trend using future climate scenario data (2037–2062) (b). The right picture (c) shows difference between the future and the past common spatial trends

**Figure 10:**
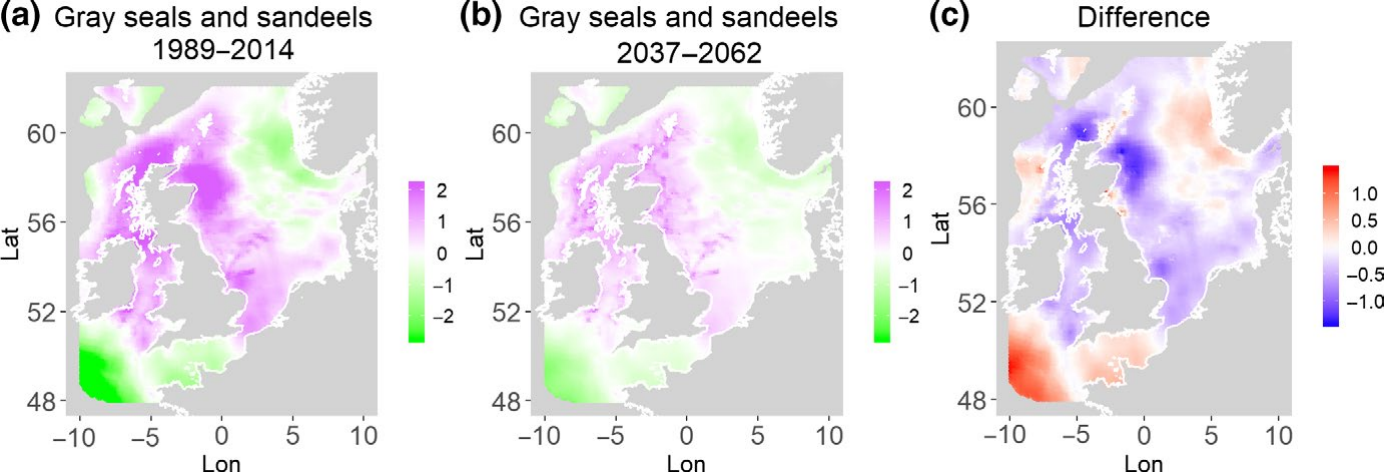
Gray seals and sandeels. Estimated present common spatial trend (1989–2014) (a) and future (projected) common spatial trend using future climate scenario data (2037–2062) (b). The right picture (c) shows difference between the future and the past common spatial trends

**Figure 11:**
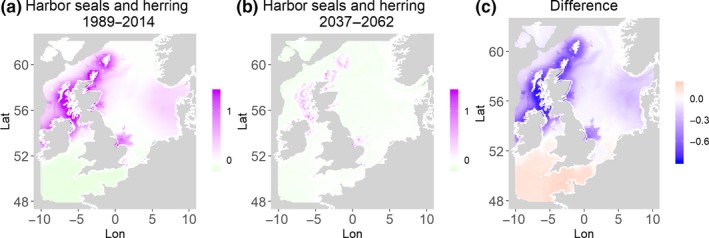
Harbor seals and herring. Estimated present common spatial trend (1989–2014) (a) and future (projected) common spatial trend using future climate scenario data (2037–2062) (b). The right picture (c) shows difference between the future and the past common spatial trends

**Figure 12:**
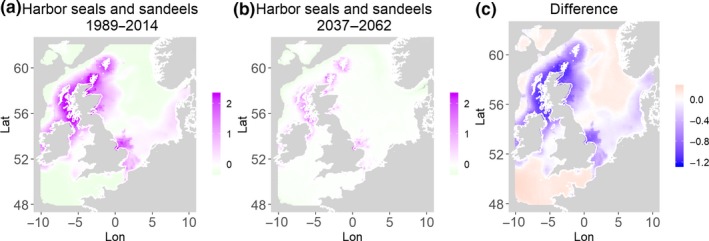
Harbor seals and sandeels. Estimated present common spatial trend (1989–2014) (a) and future (projected) common spatial trend using future climate scenario data (2037–2062) (b). The right picture (c) shows difference between the future and the past common spatial trends

**Figure 13:**
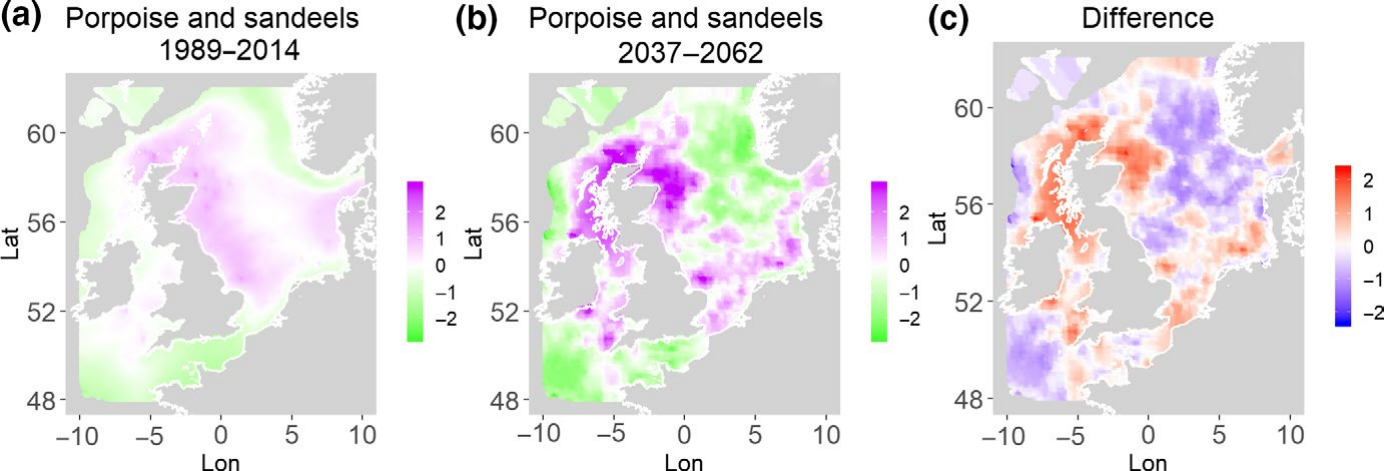
Harbor porpoise and sandeels. Estimated present common spatial trend (1989–2014) (a) and future (projected) common spatial trend using future climate scenario data (2037–2062) (b). The right picture (c) shows difference between the future and the past common spatial trends

**Figure 14:**
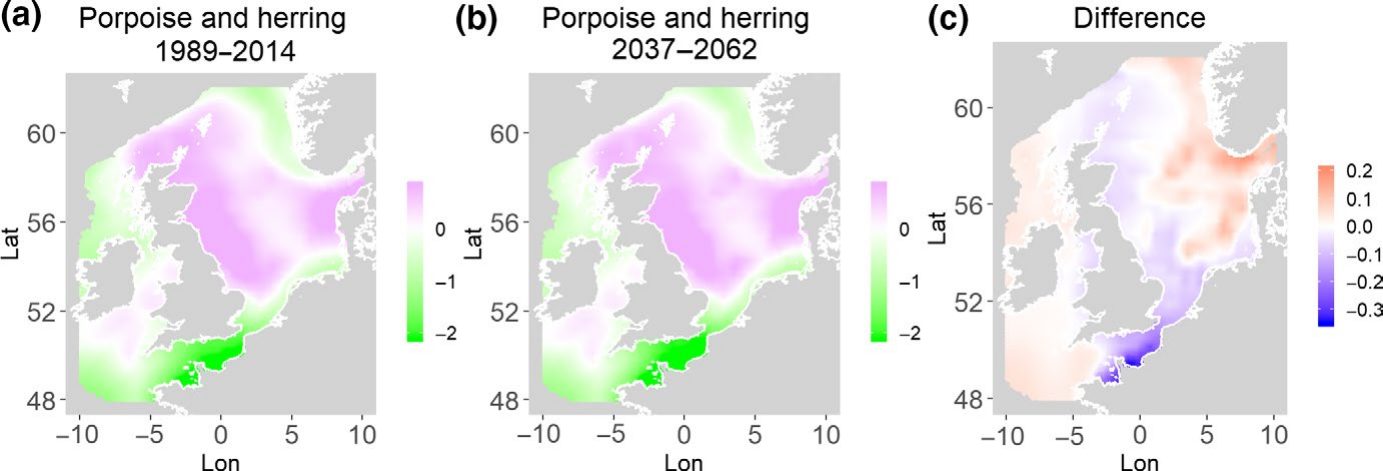
Harbor porpoise and herring. Estimated present common spatial trend (1989–2014) (a) and future (projected) common spatial trend using future climate scenario data (2037–2062) (b). The right picture (c) shows difference between the future and the past common spatial trends

The common spatial trends (Figures 1, 2, 3, 4, 5, 6, 7, 8, 9, 10, 11, 12, 13, 14) are also somewhat different from those published in Sadykova et al. (2017). This is due to the fact that we used predictive modeling in this study with the new and updated biophysical variables from the NEMO‐ERSEM models, while Sadykova et al. (2017) used descriptive analysis to provide insight into the past data. DIC‐based joint‐species model selection results can be found in Table 2. Only the best‐supported models are shown and they have DIC differences greater than the next best model, by at least 5 units. The joint‐species model selection results demonstrate that both PEA and NPP (Table 2) play vital roles in determining joint habitat preferences (for 11 and 8 appearances, respectively, out of 14 models). CHL is the next most important environmental variable showing up in nearly half (6) of all models. SP was also important (5), appearing in almost all models (4 out of 6) with herring involved. BT appeared only in 2 joint models, and as it is highly correlated with PEA, the results indicate that in the majority of cases PEA is a better explanatory variable than BT.

**Table 2:** Deviance information criterion‐based joint‐species model selection results

Model	BT	CHL	NPP	PEA	SP
Gray seals and harbor seals					
Common guillemot and black‐legged kittiwake					
Northern gannet and herring					
Northern gannet and sandeels					
Common guillemot and herring					
Common guillemot and sandeels					
Black‐legged kittiwake and herring					
Black‐legged kittiwake and sandeels					
Gray seals and herring					
Gray seals and sandeels					
Harbor seals and herring					
Harbor seals and sandeels					
Porpoises and herring					
Porpoises and sandeels					

Note

Only the best‐supported models are shown, and variables included in the best models are shaded in blue. The biological and physical variables are as follows: bottom temperature (BT), maximum chlorophyll‐a (CHL), net primary production (NPP), potential energy anomaly (PEA), and depth‐averaged current speed (SP).

### 4.3 Differences in spatial density: Ecological cost estimates

#### 4.3.1 Single species: Relative population change, Method 1

Table 3 provides the percentage overall change in (mean) population density or abundance between present and future populations in 2050 for each species across all possible joint habitat parameters.

**Table 3:** Single species. All columns show the range of values obtained by comparing a series of future (projected) and current single‐species densities

Model Single species	Relative population change (±%) Method 1	Overall percent difference Method 2	Change %decrease (hot to cold) Method 3	Change %increase (cold to hot) Method 3	Centroid distance (range km) Method 4
Gray seals	−7.2 to −3.1	10.1–57.0	0.3–12.7	0.3–10.7	2.0–7.4
Harbor seals	−14.1 to +2.4	60.1–81.9	0.1–23.9	0.0–9.2	0.9–30.4
Porpoises	−5.0 to +0.2	4.9–36.0	0.1–11.2	0.0–5.2	7.8–49.8
Northern gannet	−5.7 to +2.3	12.7–34.0	7.5–15.4	0.6–11.3	2.2–35.7
Common guillemot	−13.0 to +4.6	36.6–72.2	0.1–14.6	0.5–8.0	0.5–80.7
Black‐legged kittiwake	−3.2 to +0.8	20.9–25.8	8.8–21.5	0.1–9.0	2.2–25.2
Herring age 1	−18.5 to −7.5	20.0–48.2	0.0–3.8	0.0–2.6	5.3–34.7
Herring age 2+3	−24.0 to +4.7	74.8–82.4	1.2–45.2	7.1–27.1	44.7–119.2
Sandeels	−14.0 to +2.8	42.6–66.2	6.7–32.4	3.6–16.3	5.9–56.8

Note

“Relative population change” column shows percentage of population growth or decline relative to the current climate. “Overall percent difference” column shows what percentage of all the grids that exceed 33% changes in density in each grid cell by 2050 in relation to the present population densities. Change % decrease or %i ncrease columns show percentages of the population that will move from “hot spot” to “cold spot” (% decrease) or vice versa (% increase) by 2050. “Centroid distance” column shows range of distances between the center points of present and future population densities.

#### 4.3.2 Overall spatial percentage difference: Method 2

##### Single‐species models

Table 3 shows the range of percentages of grid cells that are projected to exceed 33% difference in density by 2050 in relation to the present densities. Harbor seals and both prey species, herring (age 2+3) and sandeels, show “very different” percentage density distributions to their overall future spatial density distributions with >67% of the grid cells having >33% differences, suggesting environmental effects are very important in determining their future distributions. On the other hand, porpoise, gannet, and kittiwake showed “fairly similar” spatial density distributions between present and future distributions, suggesting that, under the influence of only environmental variables, they will not shift their current distributions very much. The rest of the species, gray seal, guillemot, and herring (age 1), showed a range of responses from “fairly similar” to “fairly different” distributions.

##### Joint models

Table 4 shows the percentages of grid cells in the 2050 climate common spatial trend that are projected to exceed 33% difference in relation to the present climate common spatial trend. Almost all joint models showed “very different distribution” with 11 out of the 14 models significantly different in their spatial composition in the future. Therefore, according to the “thirds rule of thumb” (Berry, 2007), the joint porpoises and herring distribution is the only outcome that is “fairly similar,” while the other 2 joint models (gray seals and herring, guillemots and herring) are “fairly different” suggesting there will be a great deal of change in common spatial trends across most species in the future.

**Table 4:** Joint models

Model	Overall percent difference	Common space % decrease (−)	Common space % increase (+)	Centroid distance (km)	Direction
	Method 2	Method 3	Method 3	Method 4	Method 4
Gray seals and harbor seals	85.5	1.4	17.5	28.5	North
Common guillemot and black‐legged kittiwake	77.6	14.5	8.9	72.5	Northwest
Northern gannet and herring	95.1	14.0	0.1	44.0	Northwest
Northern gannet and sandeels	89.1	18.2	24.0	99.6	Northeast
Common guillemot and herring	63.2	0.01	9.3	32.2	Northeast
Common guillemot and sandeels	90.2	16.6	14.1	38.3	Northeast
Black‐legged kittiwake and herring	75.8	30.5	0.00	11.6	East
Black‐legged kittiwake and sandeels	91.6	42.5	1.3	98.1	Northwest
Gray seals and herring	47.8	5.4	2.9	41.7	Southeast
Gray seals and sandeels	82.2	11.4	0.4	71.4	Northwest
Harbor seals and herring	98.1	48.5	0.4	91.7	North
Harbor seals and sandeels	92.8	30.6	0.01	71.1	Northwest
Porpoises and herring	11.1	0.6	1.0	16.0	East
Porpoises and sandeels	89.1	18.5	11.2	164.2	Southwest

Note

“Overall percent difference” column shows what percentage of the common spatial trend in 2050 exceeds 33% difference in grid values in relation to the present common spatial trend. Common space % decrease or increase columns show percentages of the lost or gained common habitat areas by 2050, respectively. Distances between the center point of the estimated common spatial trend (based on positive values) in 1989–2014 and the center point of the common trend in 2037–2062 (“centroid distance” column). Forecast of moving directions of species' climate migrations (“direction” column).

The drawback of this method might be dealing with an amount of very small values or values close to zero, whose percentage change might be detected as significantly different, while the effect might be insignificant—depending on the errors of the predicted values. However, the results of the outcomes to investigate the potential issue of small values influencing outcomes concluded there was no effect (Table S1). However, one should apply this method with care when dealing with maps that consist of large numbers of small values (especially alongside relatively large values).

#### 4.3.3 Local and common space percentage loss or gain: Method 3

##### Single‐species models: Percentage of local area loss or gain

Table 3 provides the range of values for the percentage of grid cells changing from hot to cold or cold to hot spots for single‐species models across the range of joint habitat parameters. Both prey species showed the largest changes in local high‐density areas with the maximum percentage of grid changes in sandeels and herring ranging from 32% to 45% decreases (hot to cold) to 16 to 27% increases (cold to hot), respectively. The species that show the next biggest decreases are kittiwakes and harbor seals (with potential maximum losses of 22%–24% of local areas). Those species that showed the largest increases are gannets and gray seals (maximum 11%). Future (projected) differences in spatial densities from the single‐species models may be found in Figures S1.1b–S1.9b. Most of the spatial outcomes discussed above showed population decrease in the present high activity areas.

##### Joint models: Percentage of local common spatial trend lost or gained

Table 4 shows the percentage loss or gain of common trend by 2050, for the joint models. The harbor seal and herring model showed the highest decrease in common spatial trend, 48.5%, compared with the present overlap. Kittiwake and harbor seals, both coupled with sandeels, also showed a high percentage of lost common habitat areas (>30%). However, gannet and guillemot models, both coupled with sandeels, showed high increases in common spatial trends (14%–24%). The only other major gain was between gray and harbor seals, which showed they will share 17.5% more common spatial areas. The estimated common spatial trends for competing and predator–prey species can be seen in Figure 1a,b–14a,b. The purple areas (with values >0) of these common spatial trends identify the high‐density areas of the coupled species (and show positive spatial effects on the estimated joint populations). Figures 1c‐14c show differences between the future and the past spatial trends, where blue areas will be less suitable habitat areas by 2050 and red areas will be more suitable habitat areas, relative to the present habitat zones. In these joint models, herring of different ages were regarded as separate species (Sadykova et al., 2017), such that the joint models with herring and predator species essentially contained 3 species. Overall, most of the future‐projected common spatial trends showed decreases relative to the present high activity areas. Some joint models produced completely new suitable common habitat areas (kittiwake and sandeels, gannet and sandeels) and a few indicated higher species concentrations in the present high activity areas (porpoise and sandeels, guillemot and kittiwake).

#### 4.3.4 Weighted centroids and direction of change: Method 4

##### Single‐species models

Table 3 shows that the centroids of the single species may move by a range of 0.5–119 km, depending on the selected species and different biophysical variables present in the models. Both prey species showed the most significant centroid point shifts with herring age 2+3 (45–119 km) and sandeels (6–57 km). Guillemots had the widest range (0.5–81 km).

##### Joint models

Table 4 shows estimated distances between the centroid of the estimated common spatial trend (based on positive values) in 1989–2014 and the centroid of the common trend in 2037–2062. The distances vary from 12 to 164 km. Overall, 7 out of 14 models showed large shifts (>70 km) between present and future central points and 2 models showed small shifts (<20 km, porpoise and kittiwakes, both with herring) and 5 models showed moderate shifts (20–70 km). The harbor porpoise and sandeel model is projected to have the largest shift in the common trend (164 km). On the other hand, the harbor porpoise and herring model shows a small shift (16 km) in the joint trends. All models with sandeels as prey, except common guillemot (38 km), showed large changes in distance (71–164 km) of the centroid between present and future predictions. All models with herring as prey, except for harbor seals (92 km), showed smaller changes in centroid distance (12–44 km). The direction of change in the centroids was not all to the north (which might be expected due to the northward movement of temperature zones), with only the competing seals and harbors seals and herring moving directly north. In fact, all of the joint models with seabirds had either a northwest or northeast direction of change and gray seals and herring had a southeast change with porpoises and herring having a move to the east.

### 4.4 Ecological costs comparisons

**Figure 15:**
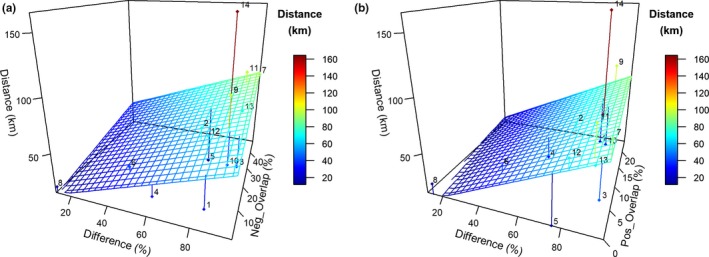
(a) Three‐dimensional plot of the regression plane relating centroid distances for common spatial trends (km), percentage difference (%), and common space decrease (%) from Table 4. (b) Same as (a) but with common space increase (%)

All four ecological cost method comparisons are not strongly correlated with each other (Figure 15a,b). For example, the kittiwake and herring model showed a low centroid movement (12 km) but a large decrease in common spatial trend (30%) and “very different distributions” with a high number of grid cells (76%) with >33% percent difference in density, indicating that low centroid movement cannot be assumed to mean nonsignificant spatial changes. Overall, there is a weak relationship between the common spatial trend percentage change and the centroid distance, but there is no relationship between any of the other combinations of costs.

### 4.5 Comparisons with other methods: RMSE and Bhattacharyya distances

A number of different statistical methods exist for comparison of spatial maps, for example, Bhattacharyya distances/affinity, root mean square error (RMSE), or Ripley's K. We used two of the common methods to compare our results with the results obtained from the selected methods.

The RMSE results are found in Figure S3, and they are strongly correlated (*r* = .64) with the results from the weighted centroids (Method 4) and moderately correlated (*r* = .51) with the results from the overall percentage difference (Method 2). The RMSE scores are high for most of the models except porpoise and herring model (in relation to the common spatial trend values), which agrees with the major findings of this paper. The Bhattacharyya distances' results are found in Figure S4. The Bhattacharyya distance method showed the largest dissimilarity (0.58) for the harbor seal and herring model, followed by gannet and herring (0.44) and harbor seal and sandeel (0.44) models. The smallest dissimilarity is shown for the porpoise and herring model (0.01). These results are in accordance with the “Overall percent difference” results (Method 2, Table 4), which also showed the highest difference for the harbor seals and herring, followed by gannet and herring and harbor seals and sandeels with the smallest difference for porpoise and herring.

## 5 DISCUSSION

### 5.1 Use of joint modeling: comparing present and future climate‐influenced distributions

The overall approach of using Bayesian joint modeling with integrated nested Laplace approximation (INLA) was highly successful and allowed investigation of a range of methods quantifying changes in mobile predator and prey and competing species distributional population overlap in projected climate scenarios. In particular, comparing present climate conditions to the projected “business‐as‐usual” climate scenario in 2050 (IPCC, 2013; Stocker et al., 2013), for a range of three species of mammals (gray and harbor seals, porpoise) and three seabirds (gannets, guillemots, and kittiwakes) with two common prey species (herring and sandeels), has shown that, even with only three physical and two biological parameters, there can be a complex array of future distributional overlaps between species (Figures 1‐14, Tables 1 and 2). Overall, the picture is not a positive one, with many population densities or abundances projected to fall (Table 3). However, even though for some species, such as harbor seals and kittiwakes, the future looks bleak in their present habitat, if projections are correct there are new future locations which, provided there are haul‐out/colony sites and appropriate sediments available, may become more conducive to overlap between predator and prey (see Table 4 and Figures 1, 2, 3, 4, 5, 6, 7, 8, 9, 10, 11, 12, 13, 14). This approach provides better understanding of the possible mechanistic linkages between the future changes to distributions of combinations of important biophysical variables that provide predators–prey overlap. The increase in the predictability of the associated relative “ecological cost” to the level of change in important species spatial overlap is what is needed to make well‐informed decisions about the spatial locations and future anthropogenic uses of our ocean spaces.

### 5.2 Model selection and uncertainty

However, there are words of caution about this approach and it must be stressed that as we used only a single future climate projection (the HadGEM2‐ES model with RCP8.5, the “business‐as‐usual” scenario) and only one prey species at a time in this study. Here, we would also like to emphasize that the data used in that paper were collected from different sources (see “Data description” section above) and present species density or abundance. Therefore, the obtained predictions for different species might not be directly compatible with each other and were compared only to themselves (present vs. future) in this manuscript. When interpreting the results, this should be taken into account. As with all modeling outputs, there are uncertainties due to stochasticity in the spatial models, uncertainty in the values of the parameters and uncertainty in the input variables (biophysical variables, which have been provided from runs of the 3D coupled baroclinic/hydrodynamic and ERSEM ecosystem models). There is additional caution with the porpoise model outputs as, stated in the “Data description” section, the input density maps contained environmental covariates. By performing the analysis on combined data from multiple years that had very different spatial density distributions, the influence of those spatial covariates was reduced (see Table S3 and “Results” section), but the influence still needs to be considered. Due to the large size of data sets and the complexity of spatial models meant that we chose to use the DIC for model comparison, which was more straightforward to compute in the INLA approach than the alternatives such as leave‐one‐out cross‐validation (LOO; Vehtari, Gelman, & Gabry, 2017). Therefore, in our results and discussions, where we are making inference about future species densities, abundances, and future common spatial trends, we urge readers to consider these as physically plausible projections, rather than an absolute prediction and the results should be used with some care. The approach in this study is focused to explore best methods of comparisons to assess future vs present fine‐scale spatial densities rather than definitive spatial distributions.

### 5.3 Comparisons and use of ecological cost methods

The use of spatial models (with INLA), combined with the four methods of estimating possible ecological costs of climate change, which is seen in the relative amount of change in the population density or abundance, allows a range of different assessments of the quantitative changes between two distributions. This approach allows analysis of continuous distributions and joint predator/prey or competitors' distributions, which provides the means to compare and quantify changes in spatial relationships. The four methods are discussed in turn below:

#### 5.3.1 Relative population change: Method 1

The method presented here allows future projections of species population density or abundance, based on different biophysical covariates, to be made. This approach provides an informative metric that can be used to make predictions of population change due to climate or other anthropogenic disturbances. This study shows that all species are projected to have overall decreases in populations in most predator–prey and competitive relationships (Tables 3 and 4) with prey species in general doing much worse than predators.

#### 5.3.2 Overall spatial percentage difference: Method 2

Among the four methods, method 2, the spatial percentage difference measure, has an advantage of using all the spatial information, such that this method can spatially represent the differences between the two distributions for any grid cell separately and allow very fine‐scale (individual colony, haul‐out site) understanding of changes in common spatial trend at local subpopulation level. It can also be summarized into one overall measure for whole population‐level comparisons. Exploring the detail for single species (Table 3), it is quite intriguing that for herring age 1 there is relatively less change (20.0%–48.2% change) as compared with drastic differences for herring age 2+3 (74.8%–82.4% change), which may be explained by herring age 1 having no significant relationship with PEA (Table 1). The age classes of herring were modeled separately because the age classes have very different distributions (Bailey, Maravelias, & Simmonds, 1998). The approach in this study suggests that this level of detail is needed for clearer understanding of the mechanisms of future change.

For joint models (Table 4), only 3 overall outcomes did not have “very different” distributions, and these were gray seal, porpoise, and guillemot. All these species clearly show or have indications (SCANS, 2016) of currently increasing UK populations (Hammond, 2017; Harris, Albon, & Wanless, 2016; Jones et al., 2015).

Gannets are also increasing in the UK (Murray, Harris, & Wanless, 2015), and although the model outcomes show a high overall percentage change in spatial densities (Table 4), this is because future predicted overlaps show increases with prey (Figures 4 and 5). Therefore, it is interesting that the four species of predators that are currently showing stable or increasing populations are predicted in this study to either have very little change or increases in the common spatial trends with their prey in 2050. This point will be picked up in the next section.

#### 5.3.3 Local and common space percentage loss or gain

Habitat loss/gain and common spatial trend measures have an advantage of identifying hot/cold spot areas and detecting significant local changes as a response to climate change (Table 4). The two species that are already of concern in European waters, the harbor seals (Jones et al., 2015) and kittiwakes (OSPAR, 2017), both show the highest losses (31%–49%) of areas of common spatial overlap for both prey species in 2050. Also, unlike some of the other predator species, neither has major increases of spatial overlap (1% or less) in other regions with either prey species. Interestingly, the minimal increases between kittiwakes and sandeels are predicted to be very localized, mostly to the west and south (Figure 8). In contrast, the other seabird species, guillemots and gannets, have much lower levels of losses of common trend with their prey (0%–18%), but both have the potential for gains in future common spatial trend, with gannets in particular having high gains with sandeels (14%–24%, respectively). However, these models must be interpreted with caution, as they will more accurately reflect losses than they will gains, since the future projections are not constrained by the presence (or lack) of suitable breeding sites and sediment type for prey.

The potentially competing seal species (Jones et al., 2015) show a high future overlap, with a predicted 18% increase with very little decrease in overlap in present areas (<2%, Figure 1). This could be due to the fact that, although both seal species have similar negative relationships with PEA, our models' estimates have quite different relationships with NPP and opposite relationships with CHL. Climate change is projected to decrease both NPP and CHL (Holt et al., 2016). Gray seals have an optimum value of NPP closer to 200 mgC m^2^ day^−1^ and are negatively related to CHL, whereas harbor seals have an increasing relationship with NPP at values >400 mgC m^2^ day^−1^ and a positive relationship with CHL (Table 1). Interestingly, the areas in the UK where harbor seals show less projected overlap with gray seals in this study (Figure 1) (i.e., low future overlap on northwest coast as compared to northeast coast) are areas that show increases in current numbers (Jones et al., 2015). Therefore, this modeling outcome suggests that the drastic population declines in the harbor seals (the smaller, less mobile species) may be due to the combination of the change in habitats, via climate change, producing both an increased overlap with a major competitor and losses in overlap with both prey species, but also suggesting possible areas without high competition and more prey overlap in the northwest.

#### 5.3.4 Weighted centroid and direction of change

The weighted centroid movement in this study requires only two reference points: weighted centroids of present and future climate for the whole UK population distributions, which, for 11 out of 14 joint relationships, showed quite significant changes in more than 75 km. The direction of change has been shown to be highly variable in this study. It is not a simple case of all prey and predators moving north. There are complex interactions between predator–prey relationships with biophysical variables and the effects of climate change, with some outcomes showing possible new locations of overlap in northwestern, southern, and eastern areas of UK waters.

However, this approach could also be used at a much finer spatial scale within the foraging areas of breeding colonies/haul‐out sites to assess local population shifts or the change in distances that species might be required to travel in response to climate change (or other anthropogenic disturbances) on a daily timescale for foraging trips. Knowledge of the change in distance, which would be required in order to better overlap with future prey species distributions, is what is missing in most current impact assessments that try to assess the population changes in displacement due to substantial anthropogenic activities (such as large‐scale offshore renewable developments that can also change the important biophysical variables for predator–prey overlap). The explicit inclusion of future predator–prey spatial overlap estimates may make a significant difference in the assessment of possible impacts of the spatial locations of offshore developments of all kinds.

#### 5.3.5 Comparison of all four ecological costs methods

The results imply that the four methods for quantifying ecological cost using joint modeling outcomes are not consistent with each other in direction or magnitude (Figure 15a,b), and therefore, we would conclude that to understand more clearly the mechanisms that drive predator–prey/competing species overlap, it is best to use all the methods in combination. First of all, this is to understand whether (1) populations are changing at high or low rates; (2) whether the density or abundance changes are occurring significantly in many or few locations (with the worrying outcomes in this study showing for most single species over 25% of areas changing and many joint models showing over 80% of areas changing more than 33% already in 2050); (3) local areas changing from good to poor habitat (hot/cold) or foraging locations (common spatial trend) may be the most obvious in highlighting the differences between species and predator–prey overlap, as a large decrease in common spatial trend was a good single metric in picking up the 2 predator species that are currently strongly decreasing: harbor seals and kittiwakes. The combination of Methods 3 and 2 for “ecological costs” (low decreases in spatial common trend together with low percentage spatial differences) was a good pair of metrics for identifying three predator species (gray seals, guillemots, and porpoise) of the four species that have currently increasing populations, with very large increases in spatial common trend (just Method 3) identifying the forth (gannets). The combination of the four methods can suggest that large centroid movement and large spatial percentage differences, together with somewhat equal gains and losses in common spatial trends, are indicative of large increases in patchiness of areas of overlap with significant new areas of habitat appearing. We would also note that we compared our methods with 2 other common methods of comparing spatial data: root mean square error (RMSE) and Bhattacharyya distances. The RMSE method is strongly correlated with the results from the weighted centroids (Method 4) and the Bhattacharyya distances' results follow in accordance with the “Overall percentage difference” (Method 2) results, which indicates that these approaches are comparable. However, the overall results suggest the use of all four methods is recommend and in particular Method 3 was found to be the single most useful quantifier of ecological costs.

### 5.4 Conclusion: methods for detecting climate change versus other anthropogenic effects

There is a great need to understand and be able to quantify the “ecological cost,” in population terms, of the projected effects of climate change, in order to allow effective assessments of trade‐offs in future marine spatial planning, at a time when there are going to be rapid increases in demands on ocean space. Spatial demands will come from large‐scale offshore renewable energy developments (Boon et al., 2018), as well as issues of siting MPAs with climate change in mind and changes in spatial fishing distributions that may occur as a displacement due to the first two activities. Joint modeling using present versus future climate scenarios allows an understanding of whether population‐level changes, driven by both changes in habitat and predator/competitor distributions, are expected, and where in space the differences in individual species densities and joint‐species common spatial trends are changing the most. This approach, combined with four methods to assess the ecological costs, has shown that there is a need to look at a range of metrics that assess the costs, since the metrics did not correlate with each other. Examining all the costs together, focusing especially on locations where there is considerable loss or gain of common spatial trend, will provide a more mechanistic understanding of which combinations of habitat variables are more important for pairs of predator–prey and competing species' spatial overlap. The results of future predictions in general show a high degree of difference in spatial density distributions for almost all combinations of species with some models predicting new areas of potential future overlap. The level of complexity of outcomes suggests that there is a further need for much more detailed and fine‐scale exploration at specific colony sites and contrasting regions (50–100 km scale) of these types of future spatial predictions.

## CONFLICT OF INTEREST

None declared.

## AUTHORS' CONTRIBUTIONS

Prof B. E. Scott, Dr D. Sadykova and Dr A. Sadykov substantially contributed to the conception and design of the work; substantially contributed to the analysis and interpretation of the data for the work; drafted and revised the article; finally approved the version to be published; and provided agreement to be accountable for all aspects of the work. Dr M. De Dominicis, Dr S. L. Wakelin, and Prof J. Wolf substantially contributed to acquisition and interpretation of data; revised the article; finally approved the version to be published; and provided agreement to be accountable for all aspects of the work.

## Data Availability

There is dedicated space on University of Aberdeen central servers where data are curated by Prof. Beth Scott. Data storage will adhere to the University of Aberdeen Records Management (University of Aberdeen, 2018a) and Information Security (University of Aberdeen, 2018b) Policies. Data are backed‐up nightly to several locations including off‐site facilities and are maintained by the University of Aberdeen Information Technology Services.
