# Supplementary material for: Ecological costs of climate change on marine predator–prey population distributions by 2050
Source: Ecol Evol. 2020 Jan 9;10(2):1069–86. doi: 10.1002/ece3.5973 (PMC6988555; doi:10.1002/ece3.5973)
Supplement: Supplementary file 1 [file ECE3-10-1069-s001.docx]

**Ecological costs of climate change on marine predator-prey population distributions by 2050 – Appendix**

**Dinara Sadykova, Beth E. Scott, Michela De Dominicis, Sarah L. Wakelin, Judith Wolf and Alexander Sadykov**

**Appendix Tables S1, S2 and Figures S1.1-S1.9, S2.1-S2.5, S3, S4**

Table S1. Joint-Model outcomes with different threshold values. Table shows overall percent difference (what percentage of the common spatial trend in 2050 exceeds 33% difference in grid values in relation to the present common spatial trend) for different thresholds. As all the common trend values are in between -3.5 and 3.5 (mostly between -2 and 2), we selected next threshold levels: 1) 0.25 (above 0.25 or below -0.25) (to cut the smallest values); 2) 0.5 (above 0.5 or below -0.5); 3) 1.0 (above 1.0 or below -1.0) and 4) >1.0 to check the high density habitat areas. Therefore, the common spatial trend values (present data) above the considered positive threshold values or below the considered negative threshold values were kept in consideration. This approach provided more information about percentage differences of the high density habitat areas (right column) and investigated whether the whole-area percentage-difference-results were influenced by small values.

| Model | No threshold | >0.25 or  <(-0.25) | >0.5 or  <(-0.5) | >1 or  <(-1) | >1 |
| --- | --- | --- | --- | --- | --- |
| Grey seals & Harbour seals | 85.5 | 77.6 | 72.4 | 74.3 | 74.3 |
| Common guillemot & Black-legged kittiwake | 77.6 | 60.4 | 50.6 | NA* | NA* |
| Northern gannet & Herring | 95.1 | 99.9 | 99.8 | 99.0 | 98.0 |
| Northern gannet & Sandeels | 89.1 | 89.4 | 91.7 | 96.7 | 96.4 |
| Common guillemot & Herring | 63.2 | 64.5 | 67.8 | 100.0 | 100.0 |
| Common guillemot & Sandeels | 90.2 | 91.0 | 89.9 | 87.5 | 87.5 |
| Black-legged kittiwake & Herring | 75.8 | 94.7 | 82.7 | 50.5 | 50.5 |
| Black-legged kittiwake & Sandeels | 91.6 | 92.2 | 93.3 | 97.6 | 97.6 |
| Grey seals & Herring | 47.8 | 38.7 | 26.0 | 8.0 | 12.2 |
| Grey seals & Sandeels | 82.2 | 84.7 | 87.3 | 92.8 | 89.3 |
| Harbour seals & Herring | 98.1 | 99.8 | 100.0 | 100.0 | 100.0 |
| Harbour seals & Sandeels | 92.8 | 99.4 | 99.4 | 98.9 | 98.9 |
| Porpoises & Herring | 11.1 | 1.0 | 0.0 | 0.0 | 0.0 |
| Porpoises & Sandeels | 89.1 | 82.7 | 77.0 | 100.0 | 100.0 |

* There were no guillemot & kittiwake common spatial trend values more than 1 or less than (-1) in the present data. However, there are future (projected) 2050 common spatial trend values above the threshold (1.0) (with 100% percent difference).

Table S2. DIC values for the DIC best joint models.

| **Model** | DIC |
| --- | --- |
| Grey seals & Harbour seals | -245,514.3 |
| Common guillemot & Black-legged kittiwake | -230,706.1 |
| Northern gannet & Herring | -160,395.9 |
| Northern gannet & Sandeels | -210,187.8 |
| Common guillemot & Herring | -188,731.7 |
| Common guillemot & Sandeels | -200,737.0 |
| Black-legged kittiwake & Herring | -190,775.2 |
| Black-legged kittiwake & Sandeels | -203,953.0 |
| Grey seals & Herring | -215,141.2 |
| Grey seals & Sandeels | -284,201.8 |
| Harbour seals & Herring | -214,285.3 |
| Harbour seals & Sandeels | -276,700.7 |
| Porpoises & Herring | -236,054.8 |
| Porpoises & Sandeels | -312,501.3 |

Table S3. Percentage of deviance explained by each predictor (bio-physical variable). The null deviance presents how well the response variable is predicted by a model that includes only the intercept. Reduced model deviance (where the reduced model is the model with only one bio-physical variable) shows how well the response is predicted by the reduced model with only this one bio-physical variable. Percentage of deviance explained by each predictor (bio-physical variable) is then calculated by the next formula: (Null deviance – Reduced model deviance) *100/Null deviance

| **Species** | **Variable** | **% deviance explained** |
| --- | --- | --- |
| Grey Seals | PEA | 16.6% |
|  | SP | 5.7% |
|  | NPP | 4.6% |
|  | CHL | 3.8% |
| Harbour Seals | PEA | 20.2% |
|  | CHL | 5.3% |
|  | NPP | 3.6% |
|  | SP | 2.8% |
| Porpoises | BT | 9.1% |
|  | PEA | 8.0% |
|  | CHL | 4.2% |
|  | SP | 1.3% |
|  | NPP | 1.2% |
| Northern gannet | SP | 11.1% |
|  | NPP | 9.0% |
|  | PEA | 8.4% |
| Common guillemot | PEA | 25.0% |
|  | BT | 23.2% |
|  | CHL | 7.5% |
| Black-legged kittiwake | PEA | 18.5% |
|  | CHL | 16.4% |
|  | NPP | 3.4% |
| Herring age 1 | NPP | 10.8% |
|  | CHL | 6.8% |
|  | SP | 0.7% |
|  | PEA | 0.2% |
|  | BT | 0.1% |
| Herring ages 2+3 | BT | 29.2% |
|  | CHL | 20.5% |
|  | NPP | 14.0% |
|  | PEA | 12.5% |
|  | SP | 2.3% |
| Sandeels | PEA | 7.9% |
|  | NPP | 0.6% |
|  | CHL | 0.6% |

**Appendix Figures**


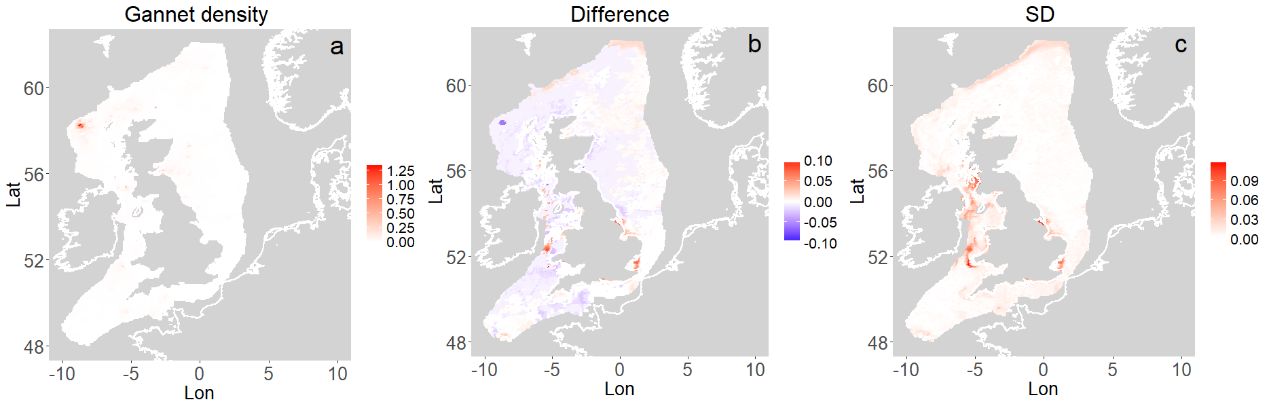
Figure S1.1 Northern gannet density map (left) (x10^2^), difference between the future (projected) density in 2050 and the current density (middle) (x10^2^) and standard deviation of the projected values (right figure). The model included net primary production (NPP) and depth-averaged current speed (SP) bio-physical habitat variables.


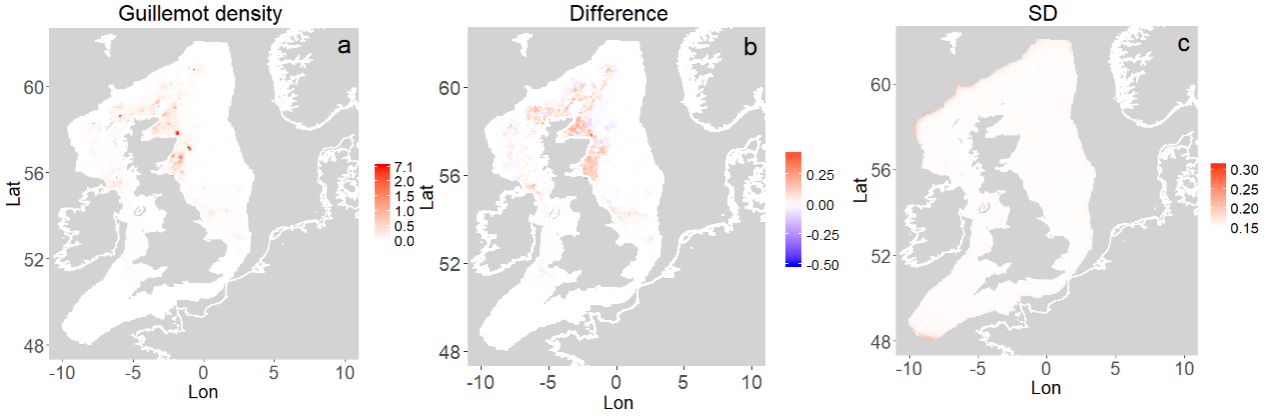


Figure S1.2 Common guillemot density map (left) (x10^2^), difference between the future (projected) density in 2050 and the current density (middle) (x10^2^) and standard deviation of the projected values (right figure). The model included chlorophyll-a (CHL) and potential energy anomaly (PEA) bio-physical habitat variables.


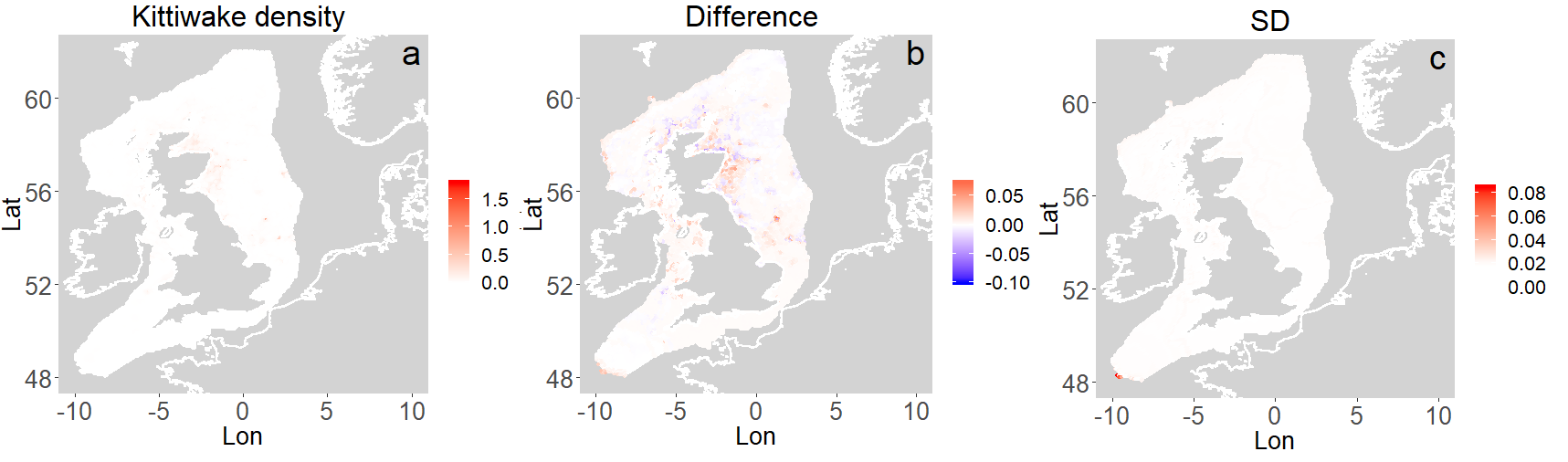
Figure S1.3 Black-legged kittiwake density map (left) (x10^2^), difference between the future (projected) density in 2050 and the current density (middle) (x10^2^) and standard deviation of the projected values (right figure). The model included chlorophyll-a (CHL) and potential energy anomaly (PEA) bio-physical habitat variables.


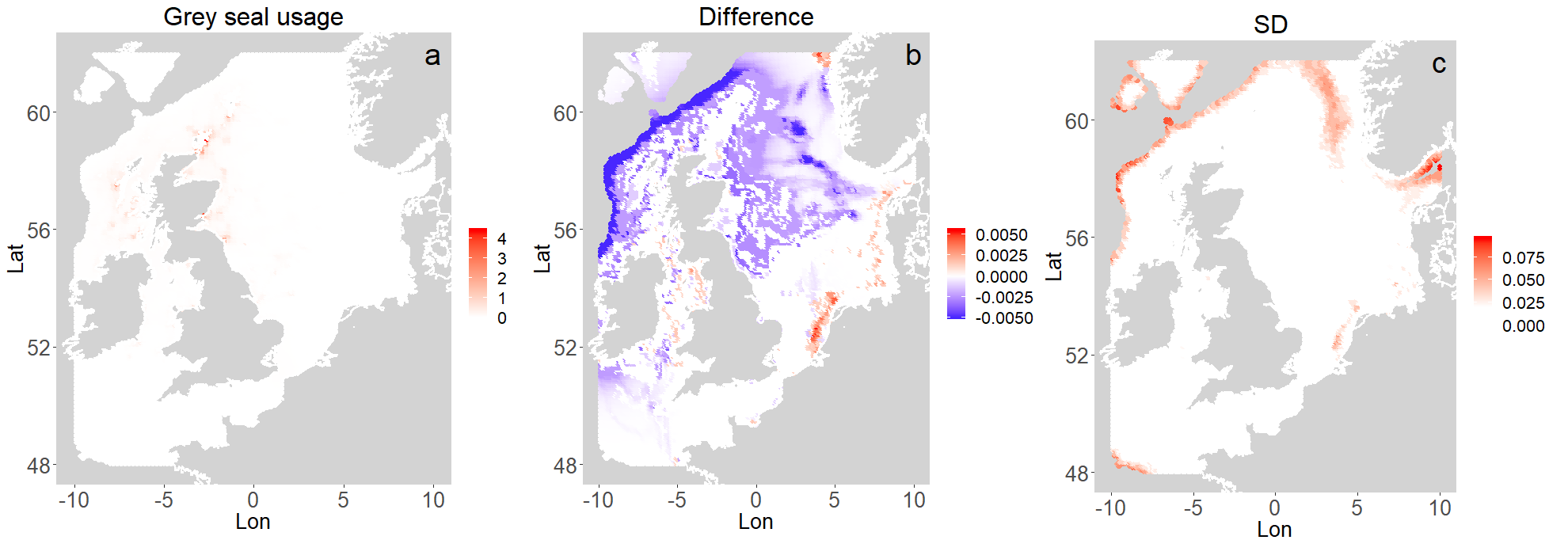
Figure S1.4 Grey seal usage map (left) (x10^2^), difference between the future (projected) density in 2050 and the current density (middle) (x10^2^) and standard deviation of the projected values (right figure). The model included chlorophyll-a (CHL) and potential energy anomaly (PEA) bio-physical habitat variables.


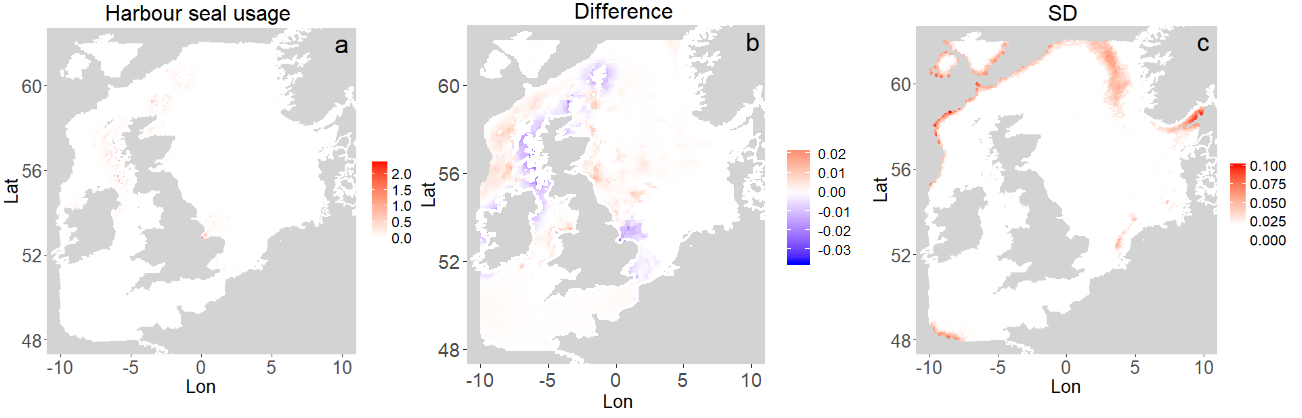
Figure S1.5 Harbour seal usage map (left) (x10^2^), difference between the future (projected) density in 2050 and the current density (middle) (x10^2^) and standard deviation of the projected values (right figure). The model included chlorophyll-a (CHL) and potential energy anomaly (PEA) bio-physical habitat variables.


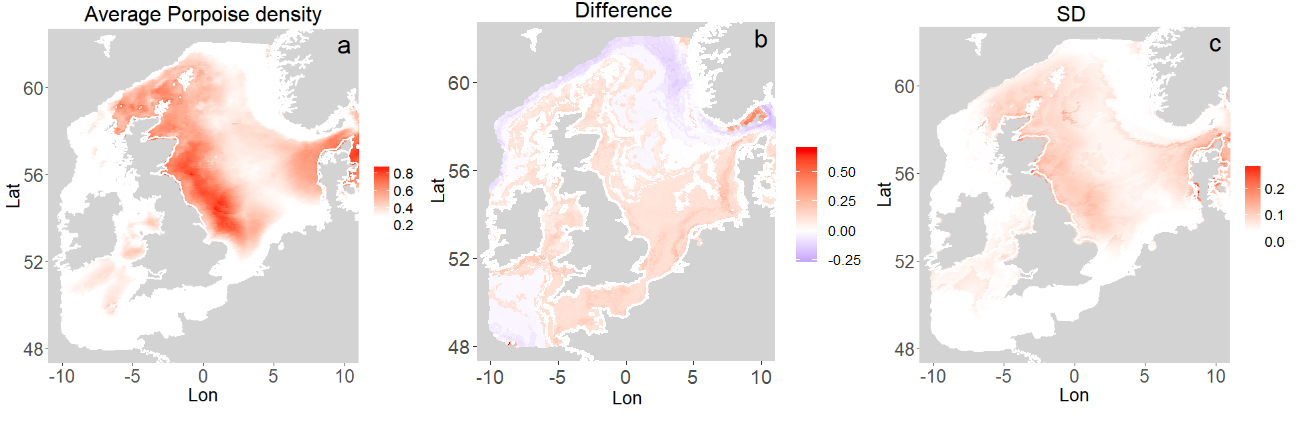
Figure S1.6 Average harbour porpoise density map (1994, 2005 years) (left), difference between the future (projected) density in 2050 and the current (averaged) density (middle) and standard deviation of the projected values (right figure). The model included net primary production (NPP), potential energy anomaly (PEA) and depth-averaged current speed (SP) bio-physical habitat variables.


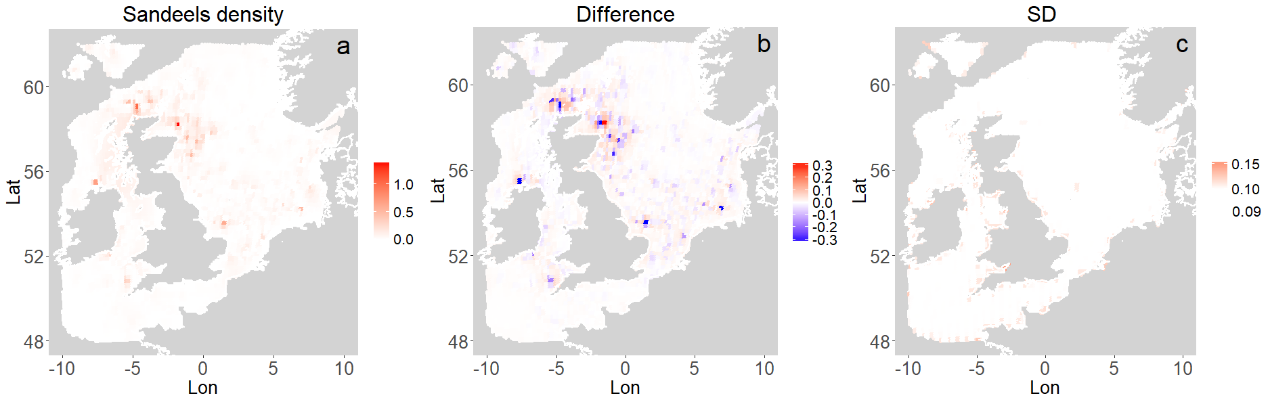
Figure S1.7 Sandeels density map (left) (x10^2^), difference between the future (projected) density in 2050 and the current density (middle) (x10^2^) and standard deviation of the projected values (right figure). The model included net primary production (NPP), potential energy anomaly (PEA) and depth-averaged current speed (SP) bio-physical habitat variables.


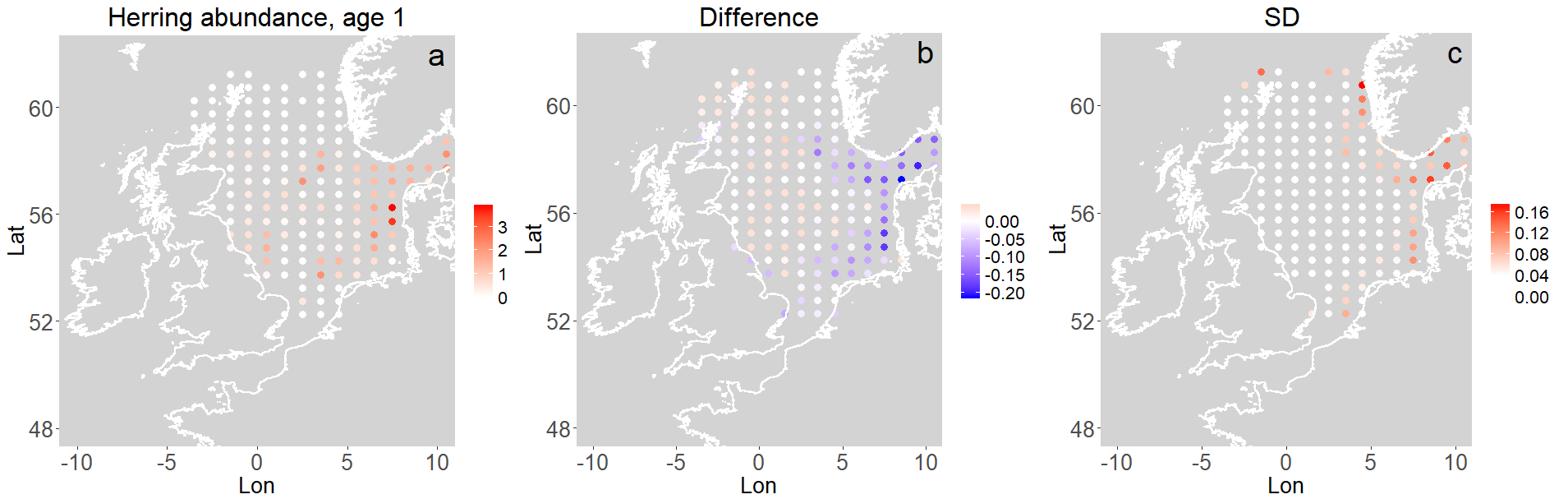


Figure S1.8 Herring abundance map for age 1 (left) (x10^8^), difference between the future (projected) density in 2050 and the current density (middle) (x10^8^) and standard deviation of the projected values (right figure). The model included net primary production (NPP) and depth-averaged current speed (SP) bio-physical habitat variables.


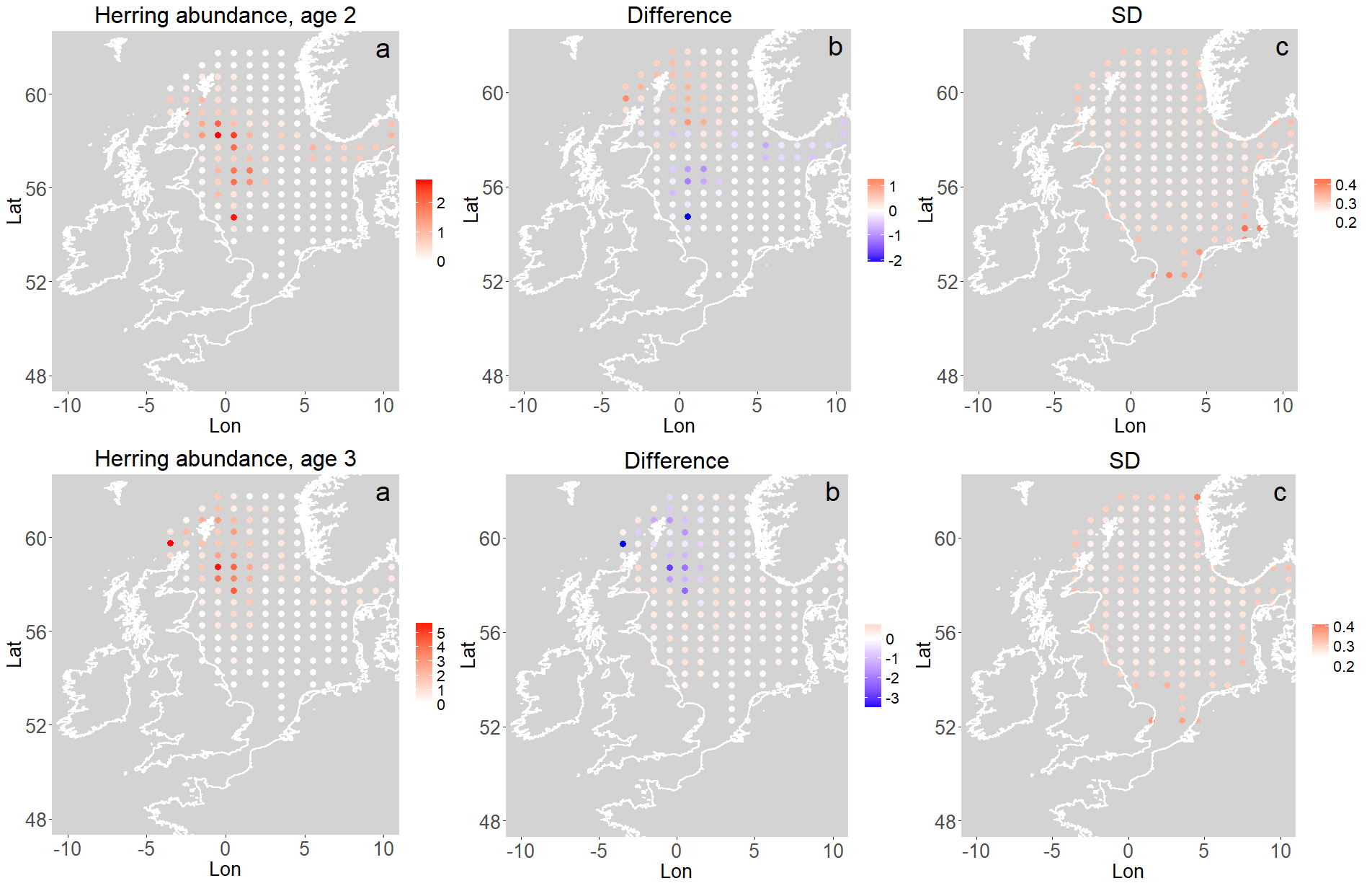
Figure S1.9 Herring abundance maps (left) (x10^8^) for age 2 (top) and age 3 (bottom); difference between the future (projected) density in 2050 and the current density (middle) (x10^8^) and standard deviation of the projected values (right figure). The model included net primary production (NPP) and depth-averaged current speed (SP) bio-physical habitat variables.


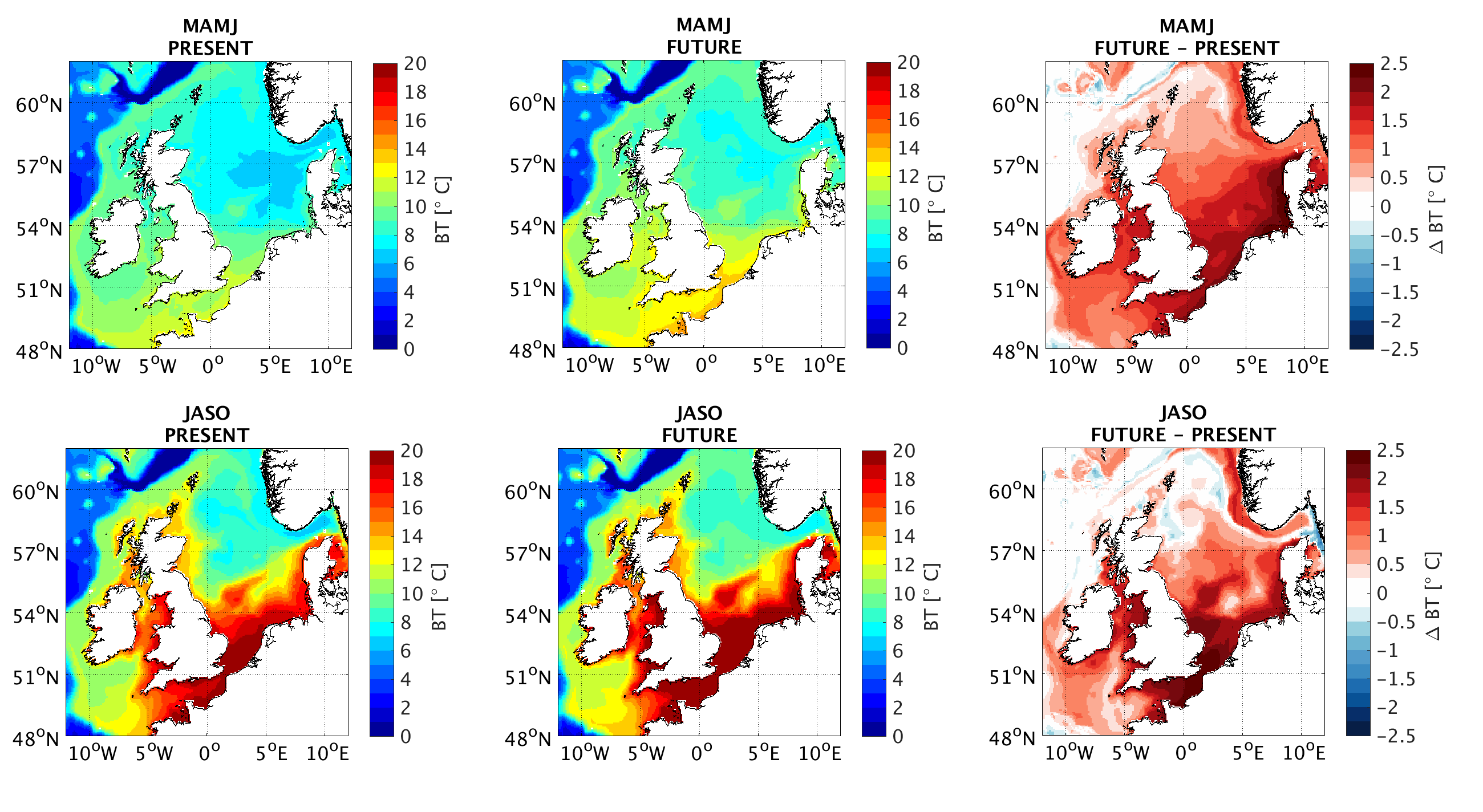


Figure S2.1 Bottom temperature (BT) (⁰C): spring season (MAMJ) (top) and summer season (JASO) (bottom). Present output (left), future output (middle) and difference between future and present outputs (right).


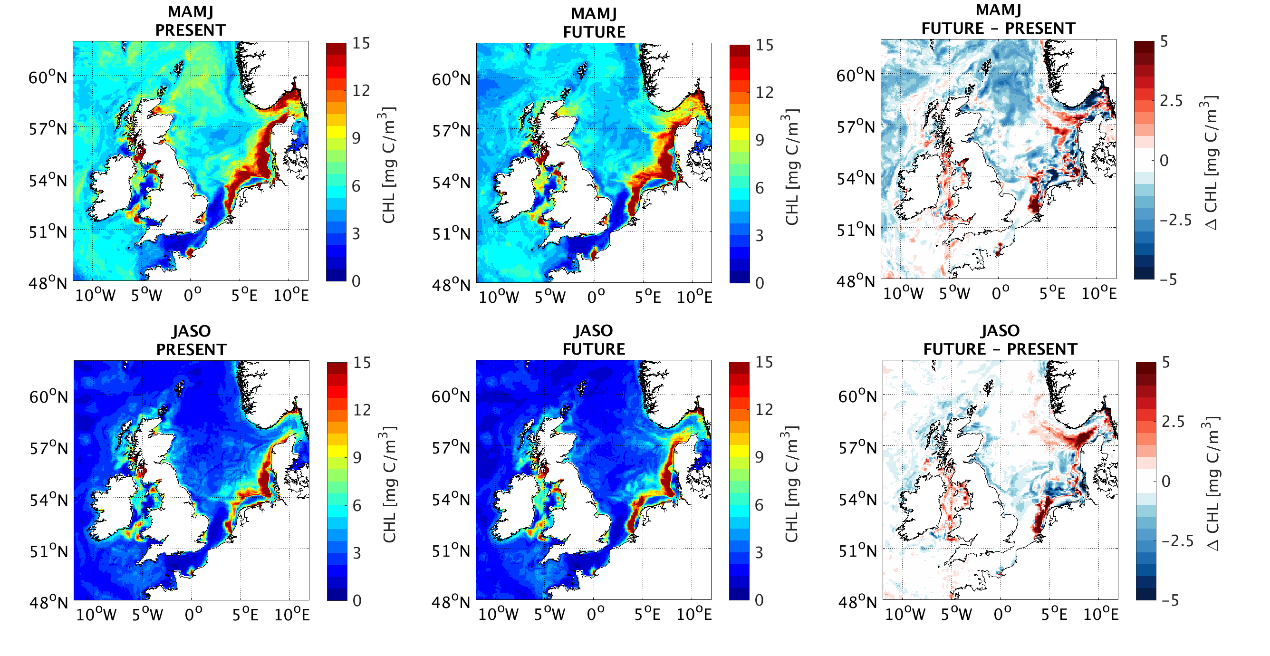


Figure S2.2 Maximum chlorophyll a (CHL) (mg C/m^3^): spring season (MAMJ) (top) and summer season (JASO) (bottom). Present output (left), future output (middle) and difference between future and present outputs (right).


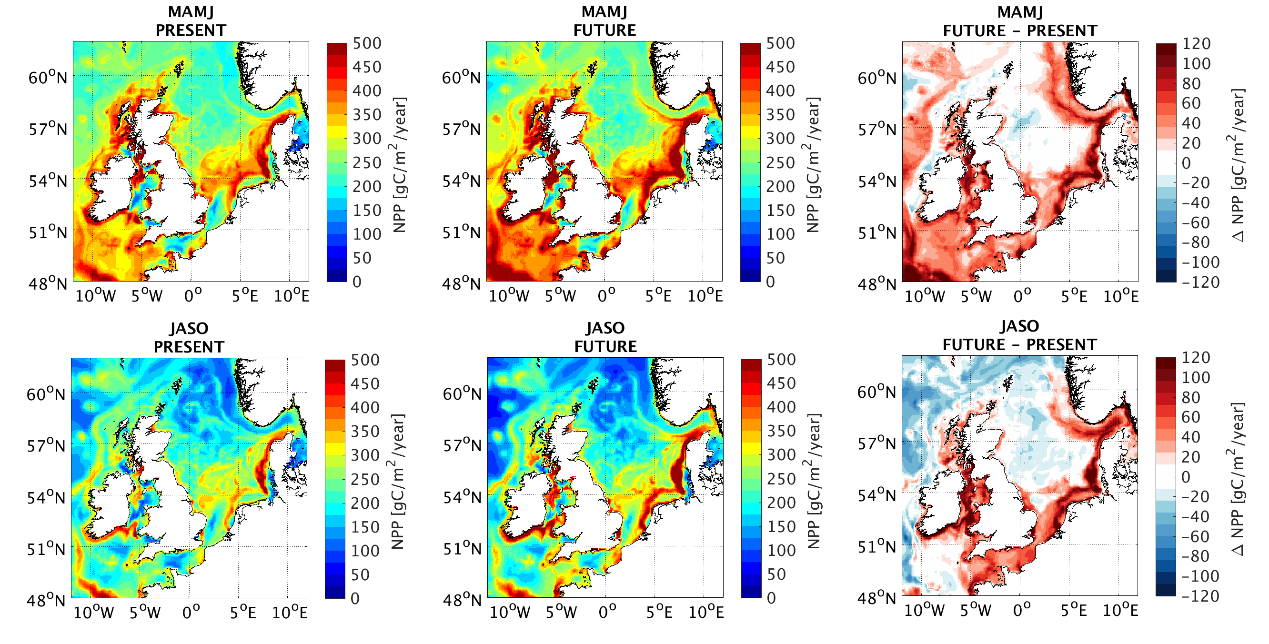


Figure S2.3 Net primary production (NPP) (gC/m^2^/year): spring season (MAMJ) (top) and summer season (JASO) (bottom). Present output (left), future output (middle) and difference between future and present outputs (right).


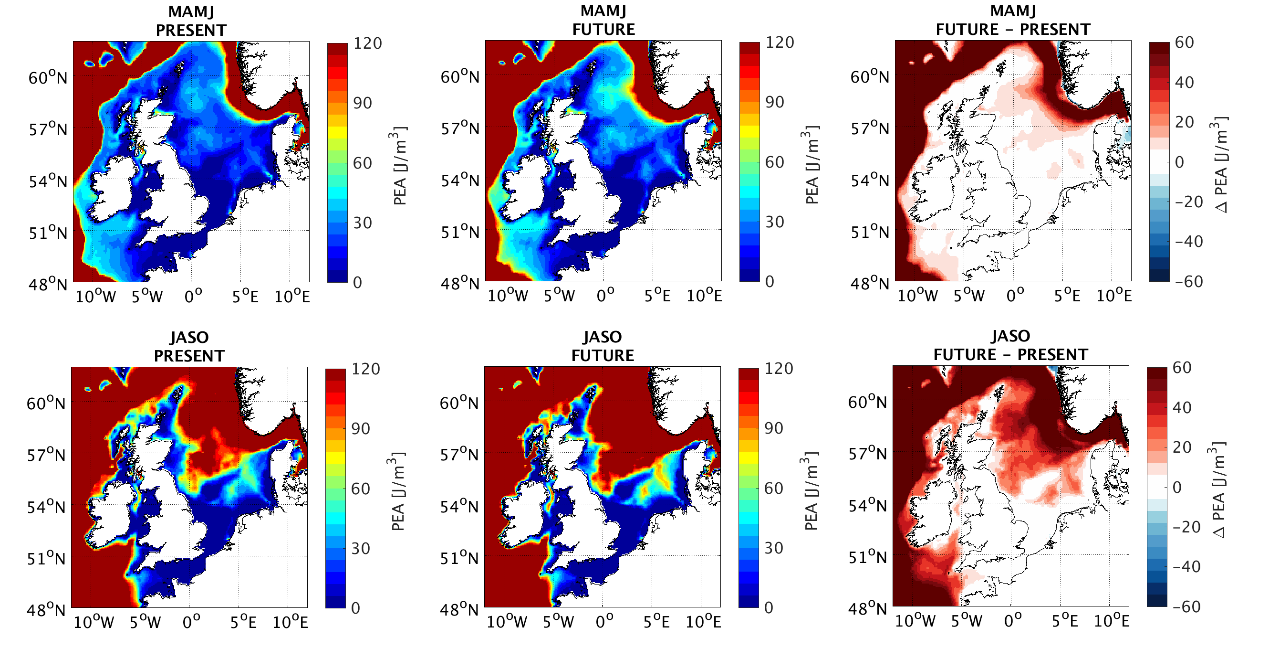


Figure S2.4 Potential energy anomaly (PEA) (J/m^3^): spring season (MAMJ) (top) and summer season (JASO) (bottom). Present output (left), future output (middle) and difference between future and present outputs (right).


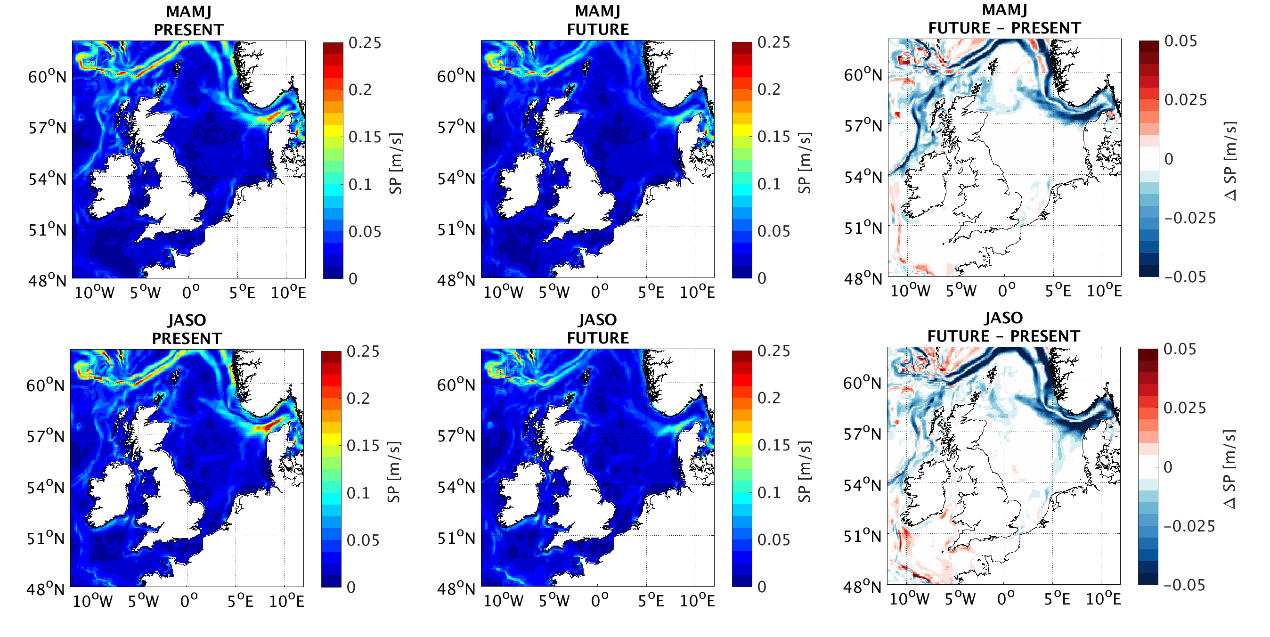


Figure S2.5 Depth-averaged current speed (SP) (m/s): spring season (MAMJ) (top) and summer season (JASO) (bottom). Present output (left), future output (middle) and difference between future and present outputs (right).


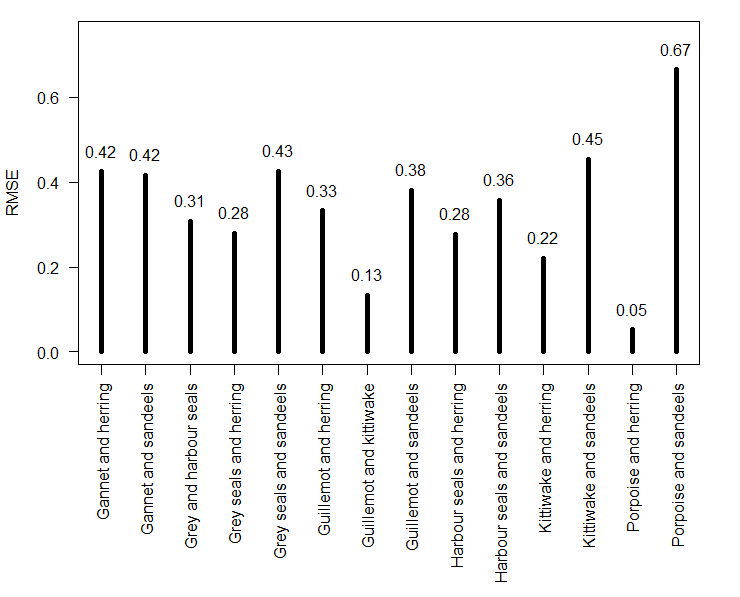


**Figure S3. Root Mean Square Error (RMSE) scores (Hyndman and Koehler, 2006) (also known as Root Mean Square Deviation), which show differences between the Future (projected) and Present common spatial trends for different models.**


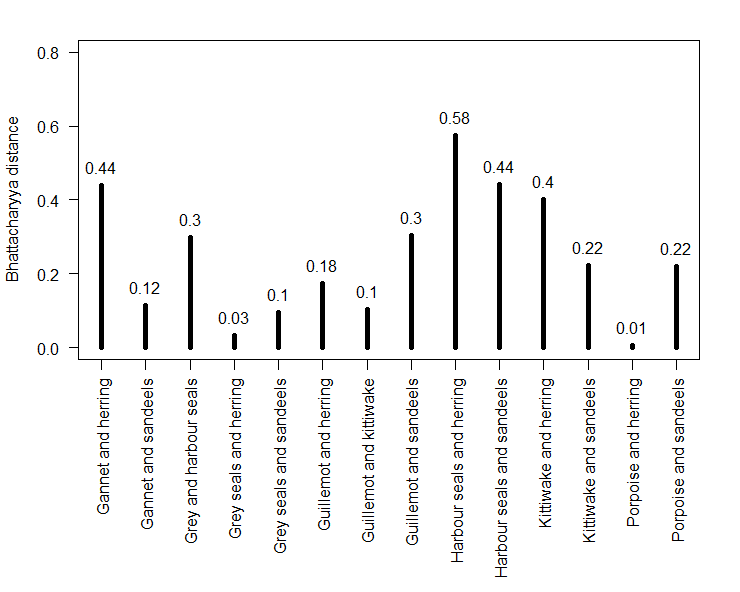


**Figure S4. Bhattacharyya distances (Bhattacharyya, 1943) (also known as ‘measure of divergence’), which reflect the degree of dissimilarity between the Future (projected) and Present common spatial trends for different models.**
